# Assessing the problem of excitation light scattering in high-viscosity jets used in serial crystallography sample delivery

**DOI:** 10.1107/S1600576725009562

**Published:** 2025-11-17

**Authors:** Stanisław Niziński, Bogdan Marekha, Jochen Reinstein, Robert L. Shoeman, R. Bruce Doak, Ilme Schlichting

**Affiliations:** a Max Planck Institute for Medical Research Jahnstr. 29 Heidelberg 69120 Germany; b UMR 7504, Institut de Physique et de Chimie des Matériaux de Strasbourg, Université de Strasbourg–CNRS, 23 Rue du Loess, Strasbourg, 67034, France; Deutsches Elektronen Synchrotron, Germany

**Keywords:** time-resolved serial crystallography, pump–probe experiments, photoexcitation, light contamination, light scattering, high-viscosity jets

## Abstract

Serial crystallography for time-resolved structural studies of light-triggered reactions often employs high-viscosity jets to deliver crystals into an X-ray beam. A potential complication is that pump light can scatter within the jet to unintentionally irradiate yet-to-be-probed portions of the jet – a problem known as light contamination. Importantly, by transporting light out of the nominal interaction region, light scattering can reduce the effective irradiation energy density experienced by the diffracting crystal. This issue, which can even jeopardize an experiment, has proven rather controversial. To provide direct insight, we performed custom femtosecond transient absorption experiments with spatially displaced pump and probe beams directed onto actual jets under realistic experimental conditions, allowing the distribution of excited molecules along the flowing jet to be mapped out explicitly. To characterize the underlying light scattering properties of commonly used jet media, the Kubelka–Munk formalism was utilized. Our results show that, in contrast to flat-cell geometries, which we found to exhibit minimal light contamination, the cylindrical geometry of jets can facilitate a degree of light spill-over. The excitation energy density loss due to the scattering is less than 30% in realistic experimental conditions. This highlights the importance of carefully selecting jet media and laser parameters to minimize light-scattering-induced artefacts when undertaking pump–probe serial crystallography experiments.

## Abstract 2

We experimentally demonstrate that the scattering-induced decrease in light intensity experienced by a crystal embedded in a high-viscosity jet is very limited in materials such as lipidic cubic phase or hy­droxy­ethyl cellulose, and therefore cannot justify the use of pump energies exceeding the linear excitation regime under realistic experimental conditions.

## 1. Introduction

Time-resolved serial crystallography pump–probe experiments can provide unique insight into reactions of light-sensitive systems. To this end, microcrystals of a protein in its dark state are excited by a well-defined light pulse (the ‘pump’) and then probed after a certain time delay by an X-ray pulse. Since many thousands of diffraction patterns need to be collected for each pump–probe time delay, rapid and efficient microcrystal delivery into the interaction region is essential. A variety of crystal delivery approaches exist (Grünbein & Kovacs, 2019 ▸; Martiel *et al.*, 2019 ▸), with high-viscosity extrusion (HVE) being highly popular since the relatively low, adjustable speed of the jet carrying the crystals into the X-ray beam results in efficient sample usage. The success of serial pump–probe experiments hinges on thorough preparation and optimization of sample delivery and on effective photoexcitation. An overly low pump laser excitation energy may jeopardize the success of the experiment owing to the low occupancies of the reacting species and low quality of the light-state electron density. Conversely, an excessively high pump laser excitation energy may result in nonlinear optical effects such as multiphoton absorption, altering the reaction pathway. The manner in which the excitation light propagates through the delivery medium plays a crucial role. Scattering of pump light can lower the excitation energy density in the interaction zone by redirecting light away from the focal point, irradiating yet-to-be-probed portions of the jet, a process known as light contamination. Knowing the extent of the scattering-induced decrease is essential for setting the correct pump laser energy, ensuring sufficiently high occupancy of the light-induced species without entering the nonlinear excitation regime. Previous reports disagree on the fraction of excitation energy density lost because of scattering in high-viscosity jets. Estimates obtained using different approaches range from highly significant (99% to 80%) (Nogly *et al.*, 2018 ▸; Claesson *et al.*, 2020 ▸; Malla *et al.*, 2025 ▸) to much more modest losses (≤ 20%) (Nass Kovacs *et al.*, 2019 ▸; Grünbein *et al.*, 2020 ▸). A recent report (Malla *et al.*, 2025 ▸) showed that the transmission through a static Super Lube grease jet decreases for very high energy densities from 76% (70 mJ cm^−2^) down to 19% (5.7 J cm^−2^) due to the occurrence of nonlinear processes such as the Kerr effect and ionization in the grease. Such effects can occur not only in the viscous matrix but also in the crystals themselves, depending on the crystal’s position within the jet relative to the gradient in the pump light. This is yet another reason why very high laser energy densities should be avoided. As common in the field, these high energies were chosen to obtain strong light-minus-dark difference electron densities. However, depending on the diffraction resolution, the data quality and in particular the magnitude of the light-induced structural changes, spectroscopy results (Do *et al.*, 2024 ▸) suggest that the expected difference electron density may be negligible. If the laser energy density is high enough, side reactions can be induced that result in mechanistically irrelevant difference electron density. For reasonable energy densities, a decrease of transmittance related to nonlinear excitation processes is not a point of concern.

Predicting the propagation of scattered light within bulk materials is nontrivial. For instance, when a dilute suspension of ∼25 nm nanoparticles is placed in a 1 × 1 cm cuvette and the centre is irradiated with a strong laser beam, significant light contamination can occur in regions of the sample that are not directly illuminated (Bavali *et al.*, 2018 ▸). However, this need not be the case once the nanoparticle concentration exceeds a certain threshold. In highly concentrated, strongly scattering suspensions, the angular distribution of scattered light becomes anisotropic, with backscattering becoming dominant. In this strong scattering regime, multiple scattering events effectively trap light within the directly illuminated region, leading to a higher probability of absorption within this region and a reduction in light contamination elsewhere. This effect is far from trivial (Bavali *et al.*, 2018 ▸). Light propagation in jets is even more complex. It depends on various factors (sample uniformity, jet geometry and diameter, refractive index of the jetting medium, presence of impurities or occasional metal or other debris particles, size and geometry of embedded crystals, *etc*.) and is not straightforward to describe in a simple way. Some effects, such as reflection from the face of crystals embedded in the jetting material, can be calculated directly assuming a specific geometry or treated as an effectively random scattering contribution. Different light loss percentages can result depending on the experimental approach, the assumptions made and the way scattering is quantified (Grünbein *et al.*, 2020 ▸). In the end, what matters is the light energy density that the crystal effectively ‘experiences’ in a real experiment with respect to the energy density determined by a reference measurement (*e.g.* using a beam profiler and laser power meter), which is almost exclusively performed in air. Making reasonable assumptions and identifying a simple, useful theoretical framework is important to bring some consistency to this topic. To date, however, this has proven to be highly controversial, notwithstanding its direct ramifications for mechanistic interpretation of a number of published ultrafast pump–probe serial femtosecond crystallography (SFX) experiments.

Here we address the issue of pump light scattering in high-viscosity jets. As a first step, we quantified the scattering coefficients of injection media commonly used for HVE crystal delivery. To jointly describe scattering and absorption phenomena in these materials we applied the Kubelka–Munk formalism (Kubelka & Munk, 1931 ▸). This is a widely accepted and relatively simple tool that delivers reasonably accurate predictions (Kortüm, 1969 ▸), sufficient for the purpose of this work. We then experimentally investigated how focused pump light is scattered in an actual cylindrical high-viscosity jet, since this cannot be estimated using simple theory. To this end, we performed a transient absorption experiment with the probe beam focus displaced along the jet axis with respect to the pump beam focus, quantifying the transient absorption signal for various displacements to experimentally determine whether the nominally dark sections of the jet upstream and downstream of the pump laser are in fact contaminated by scattered pump light. We compared these results with a Monte Carlo scattering simulation based on quantified scattering coefficients. Our investigations show evidence that Super Lube (Sugahara *et al.*, 2015 ▸) with embedded protein crystals scatters pump light significantly and is thus very prone to light contamination (Li *et al.*, 2021 ▸), while this is not the case for jets of lipidic cubic phase (LCP) and hy­droxy­ethyl cellulose (HEC) (Sugahara *et al.*, 2017 ▸). These findings agree with previous results (Grünbein *et al.*, 2020 ▸) and have obvious implications for time-resolved serial femtosecond crystallography pump–probe experiments.

## 2. Materials and methods

### 2.1. Materials

For light scattering tests, regular homogenized UHT cows’ milk with 3.5% fat (see Fig. S7 in the supporting information) and betanin extract from Sigma–Aldrich (901266) were used. Nile red dye (72485), Pluronic F-108 (542342), hy­droxy­ethyl-cellulose (09368) and thaumatin from *Thaumatococcus daniellii* (T7638) were purchased from Sigma–Aldrich. Monoolein was obtained from Nu-chek Prep. Thaumatin microcrystals (*ca* 15 µm in the longest dimension) were grown as described previously (Nass *et al.*, 2016 ▸), and microcrystals (*ca* 15–25 µm in the longest dimension) of fatty acid photode­carboxyl­ase (FAP) were grown in batch using seeding approaches (Shoeman *et al.*, 2023 ▸) and crystallization conditions similar to those described by Sorigue *et al.* (2021 ▸).

#### 2.1.1. Medium preparation, Nile red and microcrystal embedding, samples for spectroscopy

LCP was prepared by mixing 60 µl of monoolein (42 °C) with 35–40 µl of water to obtain both pure LCP and LCP for mixing with thaumatin microcrystals or Nile red, or alternatively with 35–40 µl of a solution consisting of 76 m*M* bis–Tris propane pH 7.5, 18%(*w*/*v*) Pluronic F-108 and 35%(*w*/*v*) PEG 3350 to obtain the LCP for mixing with FAP microcrystals. The solutions were mixed in two coupled Hamilton Gastight syringes until they became transparent, a process later referred to as syringe mixing. HEC viscous media were prepared by mixing 300 mg of solid HEC powder and 2 ml of either water (pure HEC matrix) or 0.8 *M* Na,K tartrate (for thaumatin microcrystals). Once the suspension started to clear (but was still granular) it was pipetted into 100 µl syringes and allowed to swell at least overnight. The HEC concentration was nominally ∼15% for thaumatin microcrystals.

Before being embedded in the various viscous materials, the protein microcrystal suspensions were pelleted by gentle centrifugation. A 12 µl volume of a rather dry crystalline pellet was syringe-mixed homogeneously with 100 µl of the viscous matrix material (LCP, HEC or Super Lube Part No. 21030). This is our typical protocol for embedding 10–25 µm sized microcrystals in LCP, yielding a good hit rate for SFX data collection using high-viscosity extrusion for sample delivery. Some LCP preparations were slightly turbid due to the unanticipated and undesired presence of micrometre-sized particles, a phenomenon described previously (Gruhl *et al.*, 2023 ▸) but not attributed there to metal particles from the syringes, as seems to be the case. This problem is covered in greater detail in Section S7 in the supporting information. These contaminated LCP samples were not used in the experiments.

Nile red was dissolved in dimethyl sulfoxide (DMSO) (20 mg in 1 ml of DMSO) and 25 µl of the solution was syringe-mixed into warm (42 °C) monoolein solution. This mixture was centrifuged to pellet any Nile red crystals that had grown. A 60 µl volume of the warm (42 °C) pink monoolein supernatant was syringe-mixed with 35 µl of 25%(*v*/*v*) DMSO. To make the LCP/Nile red/thaumatin microcrystal sample, a 10 µl volume of a rather dry crystal pellet (obtained by gentle centrifugation of the batch setups) was added to the pink Nile red LCP and syringe-mixed thoroughly.

Samples for spectroscopic investigations were prepared in 22, 100, 200 and 500 µm path length flat cells. In some cases, a small number of air bubbles formed (see for example Fig. S8 in the supporting information); however, no significant effect of these bubbles on the measured transmittance was observed.

### 2.2. Integrating sphere measurements

We employed an integrating sphere (Jasco ISV-922) coupled with a Jasco V-760 UV–vis absorption spectrophotometer. Flat demountable fused silica cells were used, with nominal path lengths of 10 µm (Starna; 20-C) and 100, 200, 500 µm (Hellma; 106-0.10-40, 106-0.20-40, 106-0.50-40). The actual path length of the nominal 10 µm cuvette was in fact 22 µm (see Section S3). Transmittance was calculated either with respect to a water sample (preliminary measurements with milk and betanin) or with respect to the same material squeezed between flat glasses with no recessed cavity (to ensure perfect refractive index match) with no embedded crystals. A more detailed description of this setup is provided in Section S2. The thaumatin-microcrystal-containing LCP and HEC samples used for measurements with the integrating sphere had a crystal density of ∼8 × 10^7^ crystals per ml (excluding small crystal debris) as determined by optical microscopy using a Hirox microscope for visualization. This approach could not be used to determine the crystal concentration in Super Lube due to very poor microscope contrast (the crystal density was >1.5 × 10^7^ crystals per ml on the basis of crystal shadow counting; it should be similar to the concentration of the LCP and HEC samples because the three samples were prepared in a similar fashion). Flat cells were mounted in the sample port of the integration sphere, always ensuring that the whole port was covered by the sample-filled part of the cell.

### 2.3. Transient absorption setup

Transient absorption experiments were performed by adapting an existing setup at the Institut de Physique et Chimie des Matériaux de Strasbourg (Liu *et al.*, 2016 ▸; Darari *et al.*, 2020 ▸), operating with a 5 kHz fundamental beam repetition rate. At the sample position, the size of the pump laser beam was usually ∼85–102 µm FWHM and that of the probe beam was ∼20 µm FWHM (the exact width along the jet depended on the particular acquisition and was always profiled before the measurement). For each experiment, the pump–probe overlap integral was calculated using the beam profiles measured before a given experiment. A TOPAS (Light Conversion) optical parametric amplifier was used to generate vertically polarized pump pulses with 515 nm central wavelength, a duration of ∼60 fs and ∼80 nJ pulse energy (filtered with a BG40 filter). For experiments with milk, 44 and 270 nJ pump energies were used. A 110 Hz chopper modulated the pump beam. A 2 mm-thick calcium fluoride plate delivered the white-light continuum used for probing, with the 800 nm fundamental beam being filtered out using a needle tip. The probe beam was split into two parts: one traversed the sample and the second served to register white-light spectrum variations. Additionally, KG5, FGS900 and NG11 filters were mounted in front of the detector. A spectrometer coupled with a Peltier-cooled CCD camera recorded probe spectra with 2 nm resolution. In the experiments performed with the running jet, the pump polarization (vertical) was set to be perpendicular to the probe polarization (horizontal), and a polarizer aligned with the probe was mounted in front of the detector (horizontal), thereby minimizing the amount of scattered pump light reaching the detector. The LCP jet was extruded at 3.5 µl min^−1^ from a capillary with 150 µm internal diameter. A rotating catcher was used to enhance jet stability (Doak *et al.*, 2023 ▸). To set the pump–probe vertical displacement of the focusing spot (along the jet), the pump focusing lens was translated using a micrometric screw. The dependence of the screw position and the displacement (Δ*x*) was calibrated before the experiment using the beam profiler camera (WinCamD with DataRay software, pixel size 4.65 µm). An exemplary calibration curve is shown in Fig. S25.

#### 2.3.1. Data analysis of the transient absorption data

For LCP samples with Nile red, a data filtering procedure was used to exclude points affected by jet instabilities. Three scans were performed for each pump–probe displacement Δ*x*. The transient absorbance Δ*A* was calculated separately for the ground state bleach (GSB) and excited state absorption (ESA) spectral ranges. All points obtained for 10 to 50 ps delays in the relevant spectral range (615–625 nm for GSB, 435–445 nm for ESA) were pooled. Those for the corresponding negative delays (−10 to −1 ps) were pooled separately – for the purpose of background subtraction. The median filtered average (with rejection ratio of 50%) was calculated for each pool separately (four pools in total for a given Δ*x*). Specifically, the median value of each pool was calculated, and then the 50% of the points in the pool lying closest to this median were selected and averaged, while the others were rejected. This allowed us to effectively eliminate points recorded when the jet was not well overlapped spatially with the probe beam. The integrated signal shown in Figs. 4 and 5 was calculated by subtracting the median filtered average obtained for negative delays (−10 to −1 ps) from that derived for positive delays (10 to 50 ps). The integrated signals obtained for both ESA (blue) and GSB (green) bands were fitted globally using a Gaussian function with shared FWHM and centre position parameters. Errors were calculated by taking the root sum of squares of two standard deviations for the selected points in each pool, for the positive and negative delay pools, respectively.

#### 2.3.2. Simulations of scattering using a Monte Carlo approach with *MCCL*

In order to validate the transient-absorption-determined light intensity profile along the jet, we simulated the light intensity within the jet using the *MCCL* Monte Carlo simulation package (Hayakawa *et al.*, 2022 ▸). We used version 8.0 of the package with fixes introduced by the developers as discussed on Github (https://github.com/VirtualPhotonics/Vts.MonteCarlo/issues/30). The package was run with 10^8^ photons, discrete absorption weighting and the Mersenne Twister random number generator. A Gaussian beam light source with 85 × 85 µm FWHM was used, approaching the infinite cylinder (representing the jet) from the side. A 200 µm-thick flat layer of material (representing air), normal to the incident beam, surrounding the infinite cylinder was defined, with absorption and scattering coefficients μ_a_ = 0.1 mm^−1^ and μ_s_ = 0.1 mm^−1^, respectively, re­frac­tive index *n* = 1, and anisotropy factor *g* = 0 (using the Henyey–Greenstein scattering function). Small μ_a_ and μ_s_ coefficients are used because non-zero values are required to tally the fluence outside of the cylinder. These coefficients are small enough compared with the layer thickness that their effect is negligible. An infinite cylinder centred within the layer is defined, with 150 µm diameter, absorption coefficient μ_a_ = 1 mm^−1^, refractive index *n* = 1.4 and anisotropy factor *g* = 0.9 (using the Henyey–Greenstein scattering function). Three cases were simulated, with scattering coefficients of the infinite cylinder set to μ_s_ = 0.1, 9.8 and 53.8 mm^−1^, representing the case of (i) no scattering, (ii) HEC with thaumatin crystals and (iii) Super Lube with thaumatin crystals, respectively. These coefficients are total scattering coefficients (*F*_DRS_ + *B*_DRS_) estimated in Table S2 in the supporting information using the DRS model (equivalent to anisotropy factor *g* = 0.9, where 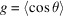 and θ is the scattering angle). The Gaussian light beam propagates along the *Z* axis, while the cylinder axis is along the *Y* axis. Fluence tally was defined with 201 × 201 × 101 bins (along the *X*, *Y*, *Z* axes), spanning a 400 × 400 × 200 µm volume. All values used in the simulation were chosen on the basis of the experimentally used jet geometry, scattering and absorption parameters determined using the Kubelka–Munk formalism, and reasonable physics assumptions.

## 3. Results and discussion

Control of the illumination conditions is essential for time-resolved pump–probe experiments. This includes knowing the pump pulse energy density (J m^−2^) impinging on the microcrystals embedded in a viscous jet. Although this seems to be a readily available parameter that can be calculated from the pump pulse energy and the beam cross-section profiled at the sample position, the situation is more complicated in reality. When impinging on a crystal embedded within the jet, the pump light can be reflected or transmitted. Upon entering a crystal, the light ray is refracted and can be further transmitted through, absorbed by or internally reflected upon leaving the crystal. These phenomena are also accompanied by diffraction [see Fig. 1(*a*) of Grünbein *et al.* (2020 ▸)]. Collectively, all of these micro-scale processes, along with any inhomogeneities in the medium, introduce a disorder in the initially focused pump beam. Intensity losses due to processes unrelated to absorption are difficult to determine experimentally because of lens effects of the cylindrical jet as well as difficulties in measuring the intensity of the transmitted light [see Grünbein *et al.* (2020 ▸) for details]. The latter issue can lead to a strong overestimation of losses due to scattering [see Nogly *et al.* (2018 ▸) and Grünbein *et al.* (2020 ▸)]. Theoretical modelling of light propagation inside the jet is complicated owing to the multitude of processes involved and the often-unknown properties of the sample itself (inhomogeneities of the jetting material, microcrystal size distributions *etc*.). We address some of these challenges using integrating sphere measurements. To quantify scattering in real-life jets, we switched to time-resolved absorption spectroscopy, which allowed us to measure the light intensity along the jet by adding dyes into the jetting material.

### 3.1. Simplistic picture of light scattering explained by the Kubelka–Munk model

HVE is often employed to deliver crystals into an X-ray beam for serial data collection. Originally developed for the injection of membrane protein crystals grown in LCP, an optically transparent material with the consistency of toothpaste (Weierstall *et al.*, 2014 ▸), the approach was quickly generalized for the injection of crystals of soluble proteins by embedding them in viscous materials such as Vaseline (Botha *et al.*, 2015 ▸), polytetrafluoroethylene (PTFE) grease/Super Lube (Sugahara *et al.*, 2015 ▸; Sugahara *et al.*, 2020 ▸) and HEC (Sugahara *et al.*, 2017 ▸). LCP, PTFE grease and HEC are commonly used injection materials for time-resolved pump–probe crystallography experiments. Consequently, it is of great interest to characterize the intrinsic light scattering properties of these materials.

To do this experimentally, one can employ a flat-cell geometry with integrating sphere detection to measure the light transmitted through a sample, including light that diverges significantly from the incident probe direction. Ultimately, however, one needs to understand the interaction of the pump light with chromophore-containing crystals in a viscous medium, which involves both absorption and scattering. This complicates determining the latter’s contribution from measurements of light transmission through the sample. To help account for both these factors, we applied the Kubelka–Munk theory, which describes light absorption and scattering properties in layers of strongly scattering materials (see Section S1).

**Figure 1:**
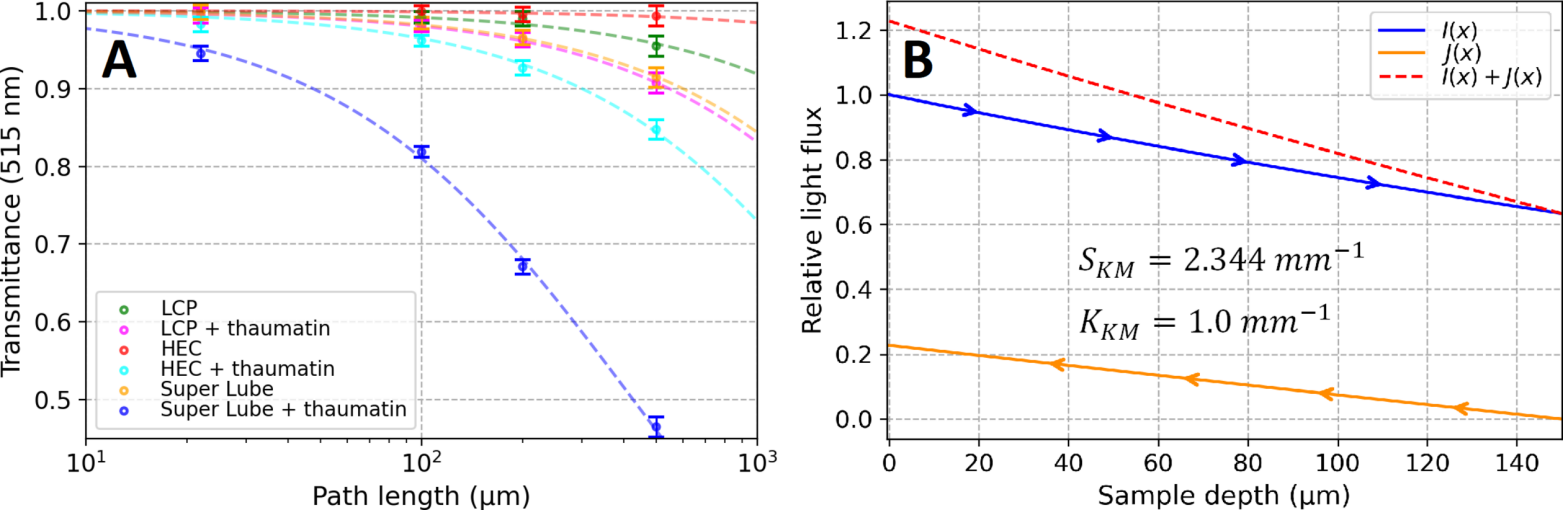
(A) Light transmission of different viscous jetting materials measured in flat cells of 22, 100, 200 and 500 µm path lengths using an integration sphere detector and fitted with the Kubelka–Munk model. (B) Exemplary forward light flux *I*(*x*) and backward light flux *J*(*x*) simulated for a 150 µm-thick sample layer using the Kubelka–Munk theory.

The Kubelka–Munk formalism (Kubelka & Munk, 1931 ▸; Kortüm, 1969 ▸; Schuster, 1905 ▸) consists of two coupled differential equations that describe two fluxes of diffuse light, one propagating in the forward and the other in the backward direction. Drawing upon known analytical solutions, we employed the analytical formula to fit transmittances obtained from the spectrophotometer coupled with an integration sphere:
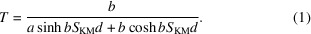
Here 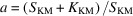, 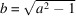 and *d* is the sample thickness. 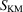 and 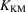 are scattering and absorption coefficients defined for diffuse light. 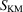 expresses the probability of a scattering event that results in reversing the propagation direction (jumping from forward- to backward-propagating mode or *vice versa*). Details are provided in Section S1. The transmittance in Fig. 1 ▸A corresponds to the foward-propagating light intensity exiting the sample relative to that entering. Within the Kubeka–Munk model formalism, the light intensity at an internal layer is the sum of forward- and backward-propagating fluxes. No light propagates backward at the last sample layer. At the first sample layer, however, the forward-propagating flux is not yet depleted, and the presence of significant backward-propagating flux results in higher light intensity than the intensity of the incident light alone. In this way, adding additional, deeper material layers increases the light intensity at shallower layers, since adding more material causes more light to be scattered backwards instead of propagating further toward infinity. This is illustrated in Fig. 1 ▸B, where both light fluxes are plotted on the basis of a scattering coefficient determined for Super Lube with embedded thaumatin crystals, combined with an arbitrary absorption coefficient. Combining the backward contribution with the forward contribution can increase light intensity inside the sample to even higher values than the incident light intensity (Fig. 1 ▸B, red dashed line).

Note that if there is no absorption (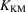 = 0 mm^−1^) the mean steady-state photon flux density within the sample is constant, regardless of the scattering magnitude (see Figs. S18A and S18B). For every ray scattered back into the sample at the last layer, there is a corresponding ray scattered out of the sample at the first layer. Other rays exhibit a similar compensatory behaviour, resulting in a constant mean light path length. Consequently, scattering simply redistributes the rays without altering the mean photon flux density. Generalized to 3D space, the mean path length travelled by light within the sample depends only on the sample geometry, not the scattering magnitude, provided that the incident light covers all possible angles of incidence (Savo *et al.*, 2017 ▸).

### 3.2. Characterization of different high-viscosity jetting materials

The light transmission of LCP, HEC and Super Lube was measured in flat quartz cuvettes of various path lengths using an integrating-sphere-equipped spectrophotometer. In all cases, the pure material and the material with embedded transparent thaumatin crystals were tested to determine how much scattering is due to the jetting medium versus the presence of crystals. By fitting the measured transmittances of LCP, HEC and Super Lube with the Kubeka–Munk model (Fig. 1 ▸A), we determined the scattering coefficients 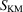 at 515 nm listed in Table 1 ▸. As shown in Fig. S12B, scattering increases – as expected – for shorter wavelengths. The addition of thaumatin microcrystals to the jetting medium significantly increased scattering in all cases. This effect is most dramatic for Super Lube (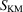 = 0.185 mm^−1^ → 2.344 mm^−1^, see Table 1 ▸). This unusual increase results from the water present in the thaumatin crystal slurry added to the Super Lube (see Section S10 for a more detailed description of this effect). Because Super Lube is hydro­phobic, very tiny water bubbles form, leading to strong scattering. A similar effect was previously observed (Claesson *et al.*, 2020 ▸); however, the conclusion that strong scattering reduces the light fluence by two orders of magnitude is incorrect, since the scattered photons do not disappear but simply continue to propagate within the material along altered trajectories. Therefore, when using any hydro­phobic matrix, the amount of water in the crystal slurry must be minimized; since this is difficult, the use of hydro­phobic jetting matrices should be avoided.

Light-flux simulations were performed using the scattering coefficient determined for thaumatin crystals in Super Lube and an arbitrary absorption coefficient 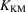 = 1.0 mm^−1^ (Fig. 1 ▸B). The results of the simulation show that in a 150 µm-thick Super Lube sample (roughly the diameter of the HVE jet used in a pump–probe investigation of photosystem II; Li *et al.*, 2021 ▸) the total light intensity, relative to the incident intensity, is reduced at the back surface of the sample and increased at the front surface as described in Section 3.1. The light intensity in the central layer of the sample corresponds to 92% of the incident light intensity. These findings would indicate that – even under conditions of strong scattering – there is no dramatic reduction of the average light intensity in the sample within the framework of the Kubelka–Munk formalism.

The scattering coefficients 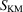 determined using the Kubelka–Munk equation (Table 1 ▸) quantify only backward scattering. The actual total scattering coefficients are substantially higher, since forward scattering predominates for protein microcrystals as their dimensions are much larger than the wavelength of visible light. We roughly estimated the total scattering coefficients (see Table S2) using a more complex scattering model, introduced in Section S5. The conclusion is that, in the strong-scattering case of thaumatin crystals in Super Lube, the collimated illumination light is effectively extinguished after 100–200 µm path length. For materials of lower scattering power, the collimated pump light penetrates even through 500 µm path length (see Section S9 for more details). The majority of the extinguished collimated mode still travels forward effectively within the sample, but as part of a disordered diffuse mode. Unfortunately, none of the applied models allow us to quantify the extent of light contamination. However, the case of thaumatin crystals in Super Lube shows that almost all light becomes diffuse while penetrating through a 150 µm-thick sample layer. The potential for light contamination in that instance is significant (in line with previous experimental findings; Li *et al.*, 2021 ▸), in contrast to the situation with LCP and HEC, which exhibit an order of magnitude lower 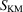 scattering coefficients.

It is generally difficult to prepare arbitrary protein crystals with and without an absorbing chromophore. Nonetheless, in Section S8 we describe how to proceed in order to characterize the absorption and scattering coefficients of a microcrystalline system of a chromophore-containing protein. Specifically, we investigated the flavin chromophore con­tain­ing fatty acid photode­carboxyl­ase crystals in an LCP matrix. We estimate 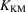
 = 0.585 mm^−1^, which reflects a realistic order of magnitude expected for an absorption coefficient of a typical chromophore-containing sample preparation used for serial crystallography experiments (see Table 1 ▸).

Certain simplifications notwithstanding, the Kubelka–Munk theory clearly can be utilized to model some aspects relevant to this work, specifically to parametrize scattering properties of materials commonly employed in HVE crystal delivery for pump–probe experiments. However, this analysis is a simplification that does not take into account (i) the cylindrical non-planar geometry of the sample medium in a jet, (ii) the finite size of the jet, (iii) the small finite cross-section of an experimental pump beam as opposed to the theoretically infinite cross-section of Kubelka–Munk theory, and (iv) the fact that the incoming light has a defined direction, whereas the scattered light is diffuse or close to diffuse. The latter drawback is partially addressed by using the more complex model discussed in Section S5. Other limitations can be only addressed by experiments investigating actual jets, such as the one performed in this work using transient absorption spectroscopy.

### 3.3. Scattering and the light contamination problem in jets

#### 3.3.1. Experimental design

In the previous sections, we quantified the scattering parameters of commonly used jetting materials (LCP, HEC and Super Lube) in a flat-cuvette geometry. This information is necessary but not sufficient to address the extent to which the pump energy impinging on the jet decreases due to scattering. We broached this issue by estimating how much of the irradiation light is transferred to diffuse modes or scattered in the backwards direction. Nevertheless, a quantitative estimate of how much light leaves the illuminated volume has not been provided. Geometry clearly plays a role. If a spatially extended object is illuminated with a spatially extended beam but probed over only a limited central region, light scattering out of the probed region into neighbouring regions can be compensated by light scattering from these illuminated outer regions into the central probed area of interest. This is clearly not the case if the illumination is tightly focused, such that the spatial extent of light redistribution (dependent on the scattering power) is comparable to or larger than the directly irradiated volume. In the case of pump–probe HVE experiments using jets, the smaller the pump laser spot and the thicker the jet, the more pronounced the problem will be. Treatment of such a geometry lies outside the Kubelka–Munk formalism and one must turn to experimental measurements conducted with an actual jet. Regardless, it is crucial to consider scattering primarily as a redistribution of light rather than a simple loss. Importantly, backscattering could additionally pose problems for high-temporal-resolution pump–probe experiments, as it can smear out the temporal resolution in strongly scattering materials, as discussed in Section S9.

**Figure 2:**
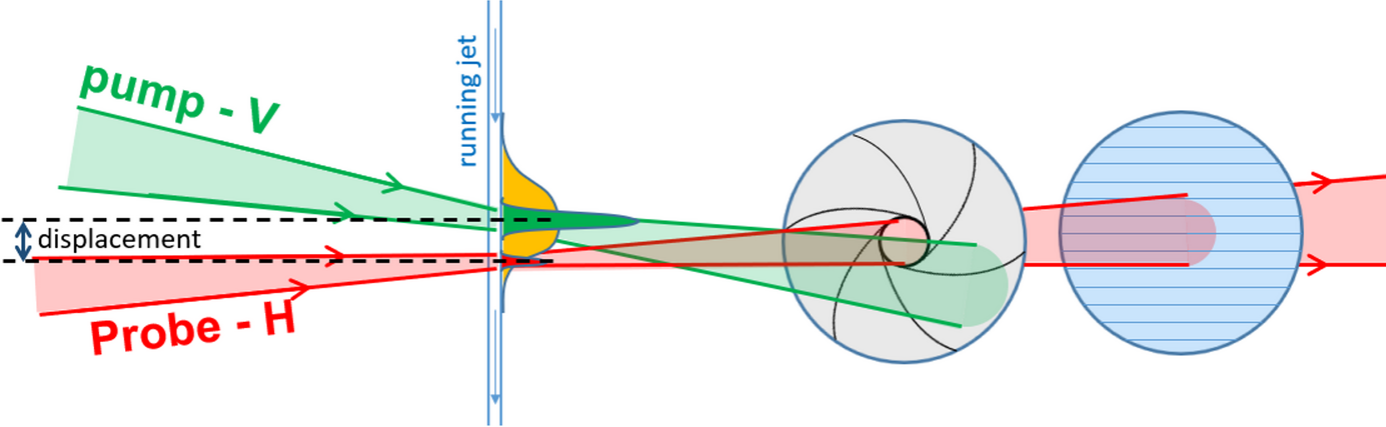
Pump–probe geometry used in the experiment to characterize scattering in high-viscosity jets. The vertically (V) polarized pump beam (green) was displaced step-wise with respect to the horizontally (H) polarized probe beam (red), so that it excited different regions located along the jet. The orange profile along the jet indicates potentially ‘leaked’ light from the directly excited sample region (green profile) due to scattering. The probe light was cleaned up from scattered pump light contributions after the interaction zone using an aperture (grey circle) and polarizer (blue circle), located before the detector. A photograph of the actual experimental setup is shown in Fig. S24.

**Figure 3:**
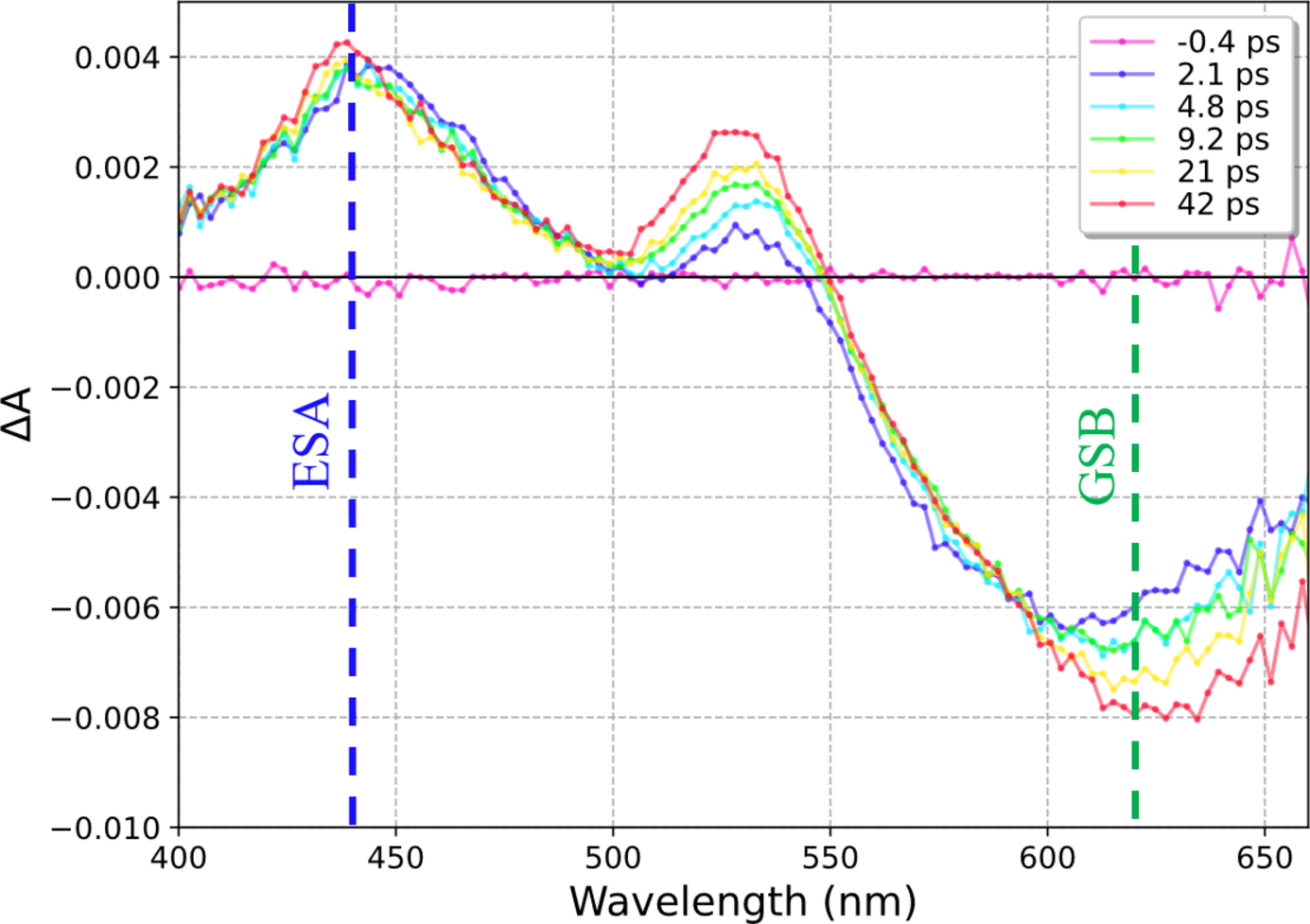
Transient absorption signal of Nile red in LCP. After excitation, there is an ESA band located at 440 nm that does not decay in our measurement time window and a stimulated emission band centred at 600–625 nm that undergoes a redshift. Both bands are used to determine the excited state population of the dye.

In order to examine the extent of pump light scattering in an actual high-viscosity jet under realistic light excitation conditions (and prior to that, in a flat-cell geometry), we performed a femtosecond transient absorption experiment where the focus of the pump laser was displaced spatially with respect to that of the probe laser to determine the spatial profile of the population of excited molecules in the sample. The experimental concept is pictured in Fig. 2 ▸. The red and green bell-shaped curves depict the transverse light intensity distributions of the incident probe and pump beams, respectively, along the jet. The orange curve is a hypothetical light intensity distribution of the pump light along the jet when broadening due to scattering is taken into account. En route to estimating the population of excited molecules, the transient absorbance Δ*A* was determined for each pump–probe spatial displacement Δ*x* using the measured intensity of the probe beam that penetrated the sample, taken at a certain delay Δ*t* and wavelength λ:
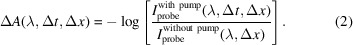


The Δ*t* value was set by delaying the probe pulse using a mechanical delay line with a retroreflector, to adjust the travel distance of the probe pulse so that it arrives before or after the pump pulse. The pump pulse excites molecules of the dye added to the viscous material of interest, and the excited state population depends linearly on the excitation energy density (provided that the pump energy is in the linear excitation regime). The measured Δ*A* signal is proportional to the population of the excited molecules. In the spectral region where the dye has an absorption band in the stationary absorption spectrum, a negative Δ*A* signal is observed since molecules are removed from their ground state (ground state bleach, GSB, see Fig. 3 ▸). A positive Δ*A* is observed in spectral regions where the excited molecules absorb light (excited state absorption, ESA, before they decay back to the ground state). The magnitude of both Δ*A* bands can be determined in our experiment to obtain direct insight into the pump light intensity distribution within the sample. Without any scattering effects, a non-zero Δ*A* signal is expected only when the probe traverses regions of the sample that were directly irradiated by the pump pulse. The pump beam was focused to a spot size close to those routinely used in serial pump–probe experiments.

The pump and probe lasers were both focused onto the jet axis (or the cuvette plane), and their intensity profiles were measured using a beam profiler positioned at the same place as the jet/cuvette. The overlap integral between the pump and the probe was calculated as a function of pump displacement Δ*x* according to the following equation:
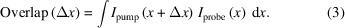


In the low-absorbance limit, this integral represents the average of the expected signal at various probed sample positions, weighted according to the fraction of the probe beam at those positions. A Δ*A* curve that is broader than the overlap integral curve of the probe and pump indicates that scattered pump light has irradiated regions of the sample that were not directly targeted by the pump beam, resulting in light contamination of nominally unpumped portions of the sample.

#### 3.3.2. Light scattering in a flat-cell geometry

The transient absorption experiments in flat cells (optical path length 200 µm) were performed first with rhodamine B dye in water and milk, respectively. Despite the fact that milk is a strongly scattering medium, we observed negligible broadening of the Δ*A* profile compared with that obtained in water and with respect to the pump and probe profiles (Fig. S26), regardless of the excitation energy density used.

Analogous flat-cell experiments were performed on viscous samples consisting of LCP with Nile red mixed in to yield a Δ*A* signal (Fig. 3 ▸ shows the spectral evolution associated with this dye). Nile red is a lipophilic dye that is routinely used as a fluorescent probe in biological systems because of its biocompatibility and large Stokes shift, and it has an absorption maximum in the 490–530 nm range in most nonpolar solvents (Gilani *et al.*, 2012 ▸; Gajo *et al.*, 2024 ▸; Krishna, 1999 ▸). It has a lengthy excited state lifetime of a few nanoseconds that, together with good solubility/stability in LCP (Deshpande *et al.*, 2014 ▸), makes it an excellent choice for this experiment. Analogous experiments with Super Lube were unsuccessful owing to a strong colour shift of Nile red from raspberry red to orange due to strong solvatochromism (Zakerhamidi *et al.*, 2012 ▸). Moreover, since Super Lube with thaumatin crystals exhibits very high scattering, it is exceedingly difficult to obtain a usable signal when using standard transient absorption spectroscopy setups.

**Figure 4:**
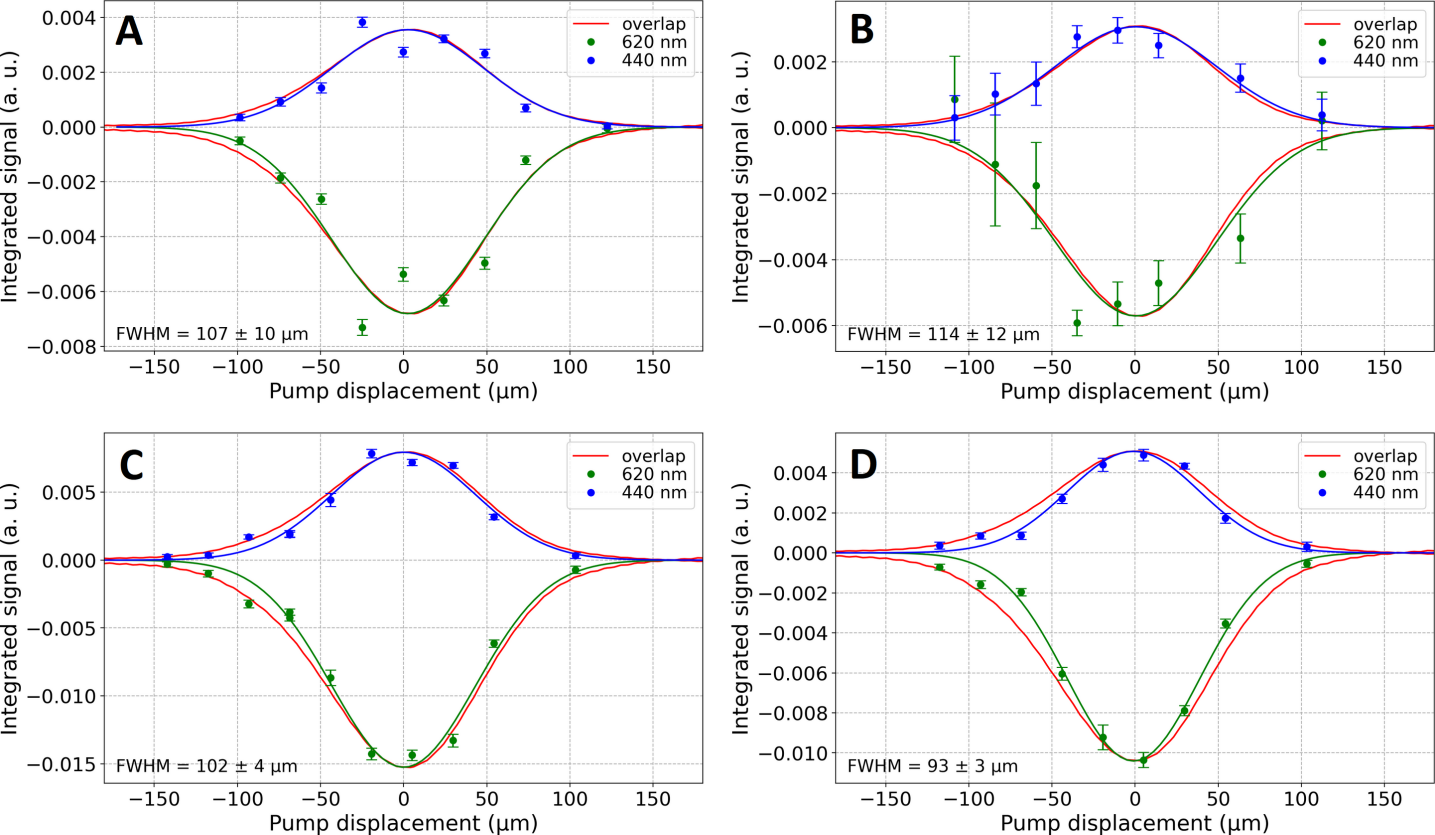
Transient absorption profile in a flat 100 µm cell. (A) LCP + Nile red, (B) LCP + Nile red crystals, (C) LCP + Nile red + thaumatin crystals, (D) LCP + Nile red (repeated sample preparation for comparison with figure A). The peak pump energy density was ∼0.7 mJ cm^−2^. The ESA band was averaged (with mean filtering) in the 435–445 nm range and the GSB band in the 615–625 nm range. Δ*A* was averaged (with mean filtering) in the 10 to 50 ps Δ*t* range, while the background signal was averaged in the −10 to −1 ps Δ*t* range. Continuous green and blue curves are Gaussian fits. The pump spot size along the jet was 102 µm and the probe spot was 20 µm (FWHM). The pump–probe overlap integral (red curve) was normalized to the green and blue curves for easier comparison.

We tested LCP with and without thaumatin crystals, in both cases with the addition of Nile red dye. Static flat cells with a 100 µm optical pathlength were used. Fig. 4 ▸ shows the Δ*A* profiles determined as a function of pump displacement (green and blue curves) superimposed with the pump–probe overlap integral (red curve). We observed no significant broadening of the Δ*A* curve with respect to the pump–probe overlap. This is in agreement with the integration sphere measurements and the low scattering coefficient obtained for thaumatin crystals in LCP (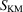
 = 0.204 mm^−1^, Table 1 ▸). All Gaussian fits resulted in 93–114 µm FWHM widths. Even in the case of LCP with unintentionally crystallized Nile red and LCP with thaumatin crystals there is no significant Δ*A* profile broadening (Figs. 4 ▸B and 4C, respectively).

#### 3.3.3. Light scattering in a flowing viscous jet

Finally, we performed transient absorption measurements in viscous jets, displacing the pump versus the probe focusing point along the jet axis (as depicted in Fig. 2 ▸). This allowed us to determine how far the scattered light might travel along the jet, reducing the pump energy density in the interaction zone and potentially resulting in light contamination. The behaviour of the LCP/Nile red sample extruded from a jet nozzle is totally different from that of the same sample enclosed in a static flat cell (described above), which is due in large part to the cylindrical geometry and intrinsic flow and fluctuations of the jet. In a jet, internally reflected light can propagate only up or down the jet and can be trapped within the jet via total internal reflection, as in an optical fibre. By contrast, the sample in a cuvette is enclosed by glass possessing a comparable refractive index, which prevents total internal reflection at the sample–glass interface. For the jet, because the refractive index of the extruded viscous material is significantly higher than that of the surrounding air or helium atmosphere, light rays propagating nearly parallel to the jet axis undergo total internal reflection.

**Figure 5:**
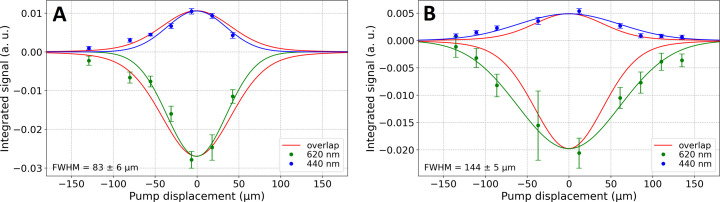
Transient absorption profile in the viscous liquid jet extruded from a 150 µm-inner-diameter capillary. (A) LCP + Nile red (with some Nile red crystals), (B) LCP + Nile red + high concentration of thaumatin crystals. The peak pump energy density is ∼1.2 mJ cm^−2^. The ESA band was averaged (with mean filtering) in the 435–445 nm range and the GSB band in the 615–625 nm range. Δ*A* was averaged (with mean filtering) in the 10 to 50 ps Δ*t* range, while the background signal was averaged in the −10 to −1 ps Δ*t* range. Continuous green and blue curves are Gaussian fits. The pump spot size along the jet was 85 µm and the probe was 20 µm (FWHM). The pump–probe overlap integral (red curve) was normalized to the green and blue curves for easier comparison.

The Δ*A* profiles determined in high-viscosity jets (Fig. 5 ▸) differ significantly from those obtained from 100 µm cuvettes. While LCP with Nile red shows a comparable Δ*A* profile along the jet axis (83 µm FWHM, Fig. 5 ▸A) to that expected from the probe/pump overlap profile (99 µm FWHM, Fig. 5 ▸A), adding a high concentration of thaumatin crystals to LCP with Nile red carrier causes a significant broadening of the Δ*A* profile, reaching 144 µm FWHM. Let us assume that the pump light (focused to 85 µm FWHM, corresponding to 99 µm of the red curve in Fig. 5 ▸) was redistributed to a 45% broader profile (99 µm → 144 µm in Fig. 5 ▸B). This would correspond to a decrease of ∼30% in the effective light intensity at the pump focal point if one assumes a full redistribution of the pump light along the jet, in accordance with the mean light path length invariance mentioned before (Savo *et al.*, 2017 ▸). Nonetheless, that invariance is strictly valid only under isotropic illumination, which is obviously not the case here. If in fact the mean path length increases with scattering, the integrated light intensity along the jet will increase. Our experiment allows us only to compare the Δ*A* profile width with the beam profile width determined using the beam profiler. Since their amplitudes are not directly proportional to each other, however, we cannot compare the integrated light intensities along the jet ‘with and without’ scattering. If the mean light path length increases with scattering, the scattering-induced loss of photon flux density in the pump focus calculated above (∼30%) is overestimated.

The difference observed between flat-cell and jet geometries could also be a consequence of crystal concentration (unlikely, but we cannot exclude this), and/or the cylindrical jet geometry may enhance the propensity for light scattering (optical fibre effect). Note from Table 1 ▸ that the scattering coefficient determined for LCP with thaumatin crystals (0.204 mm^−1^) is much lower than that for Super Lube with thaumatin crystals (2.344 mm^−1^). In view of this, it is not surprising that Li *et al.* (2021 ▸) encountered severe light contamination in their pump–probe experiment on PSII crystals embedded in Super Lube.

#### 3.3.4. Monte Carlo simulations in jet geometry using *MCCL* package

**Figure 6:**
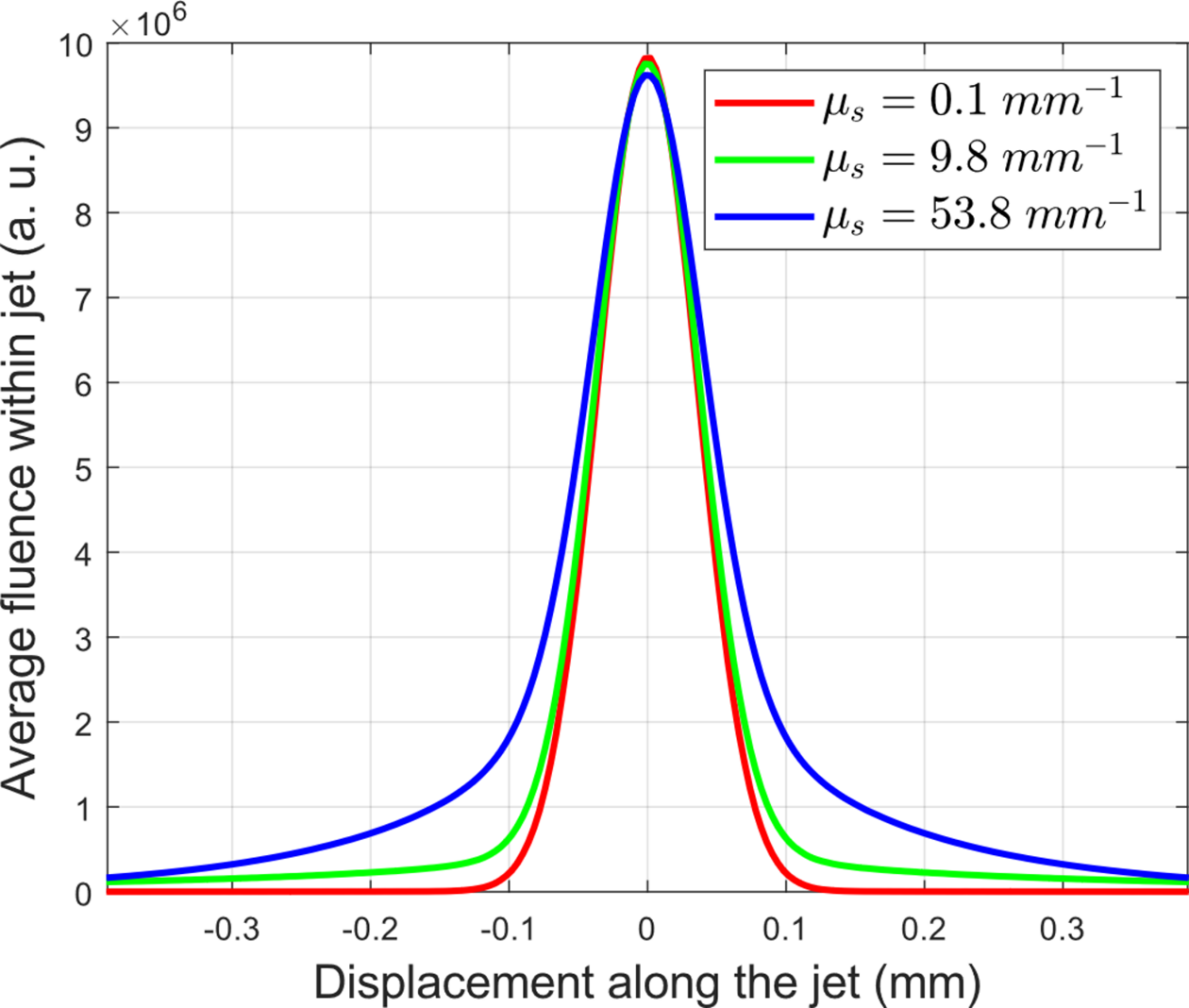
Simulated laser pump fluence along the jet axis for selected scattering coefficients. The cylinder, representing the jet, is sliced into discs, and the average fluence within each disc is plotted. The blue curve represents the scattering magnitude of thaumatin crystals in Super Lube, while the green curve represents thaumatin crystals in HEC. The red curve, simulated with negligible scattering coefficient, is a reference. Note that μ_s_ is the total scattering coefficient (taken from Table S2).

For the sake of completeness, we cross-validated our data using Monte Carlo scattering simulations, which allow visualization of light fluence under various scattering conditions. Fig. 6 ▸ shows the average light fluence within an infinite cylinder (representing the jet), simulated for cases of negligible scattering, medium scattering (thaumatin crystals in HEC) and high scattering (thaumatin crystals in Super Lube). These simulations demonstrate that, across a wide range of scattering coefficients, the light intensity within the jet at the focal position does not decrease substantially. However, high scattering results in significant light contamination, manifested as broad light distribution tails along the cylinder. Note that the fluence at a given cylinder slice is not uniform due to the lensing effect; nonetheless, moderate scattering helps to compensate for this, making the fluence slightly more homogenous (see Figs. S28–S30). Under strong scattering conditions, the fluence of the laser beam increases upon entering the jet, but less light can actually propagate through it because of ‘detouring’. The results of these simulations confirm that, in the used geometry, the integrated light intensity along the jet increases (Fig. 6 ▸), indicating that 30% loss of photon flux density is probably an overestimate (see Section 3.3.3) and that the actual loss is much smaller.

## 4. Conclusions

We have addressed the scattering of excitation light in time-resolved jet-based serial crystallography by considering the contributing factors. In a first step, we calculated the intrinsic effect of light scattering along the axis of the incident light. Use of a UV–vis spectrophotometer coupled with an integration sphere allowed us to capture almost the entire forward-propagating light flux, resulting in a well-defined experiment with results that can be inserted into the Kubelka–Munk equations to establish a picture that is simple and easy to grasp. We conclude that for weakly scattering materials such as LCP and HEC, with or without embedded crystals, light can effectively propagate through any relevant sample thickness. For Super Lube embedded with crystals, in marked contrast, light becomes fully diffuse after penetrating only about 150 µm. One consequence of the concomitant back-scattering flux is that the decrease of light intensity in the deeper layers of the sample is accompanied by an increase of light intensity in the front layers.

To investigate pump light scattering under actual experimental conditions of serial crystallography experiments we developed a custom experiment to conduct measurements on viscous jets identical to those employed for sample delivery. We measured how far from the irradiation spot the scattered light propagates along the jet, directly addressing whether scattered pump light can excite portions of the sample that are not directly illuminated. In weakly scattering jet media, namely those that scatter comparably to LCP (such as HEC), our experiments demonstrate that the light intensity distribution broadens only to a limited extent along the jet. For LCP with a high concentration of thaumatin crystals, on the other hand, we found that the light intensity distribution is broadened by roughly 45% (Fig. 5 ▸B), which corresponds to a decrease of ∼30% in excitation energy density. Monte Carlo scattering simulations demonstrate that real light loss is likely to be much closer to zero. The value of 30% should therefore be considered an upper limit for the decrease in energy density under all realistic conditions when using LCP, HEC or Super Lube with thaumatin crystals, or comparably scattering materials. A major consequence of this conclusion is that light scattering cannot in general be invoked to justify extremely high laser power densities for photoexcitation in pump–probe experiments. Scattering simply does not suffice to reduce the effective pump intensity from a nonlinear into a linear optical regime.

We conclude that extraordinary care must be taken when planning jet experiments with a highly scattering jetting medium such as Super Lube. An appropriate combination of jet speed, jet thickness, crystal size distribution and crystal concentration is essential to avoid the negative consequences of significant pump light spill-over into regions of the sample that are not directly targeted. For efficient photoexcitation it is crucial to use crystals with sizes matching the light penetration depth. Backward-scattered light in strongly scattering media, particularly in thick jets, may degrade the time resolution and so pose challenges for high-temporal-resolution experiments. Further investigation is needed to fully characterize this effect. Overall, it is advisable to select jetting materials with very low scattering properties – such as LCP or HEC – in order to preserve the initial known pump laser energy density and to reduce the risk of light contamination.

## 5. Related literature

The following additional literature is cited in the supporting information: Aernouts *et al.* (2015*a* ▸), Aernouts *et al.* (2015*b* ▸), Duntley (1942 ▸), Esatbeyoglu *et al.* (2015 ▸), Jääskeläinen *et al.* (2001 ▸), Postelmans *et al.* (2020 ▸), Ryde (1931 ▸), Ryde & Cooper (1931 ▸), Silberstein (1927 ▸), Stocker *et al.* (2017 ▸), Strack *et al.* (2003 ▸) and Wendel *et al.* (2015 ▸).

## Supplementary Material

Supporting information file. DOI: 10.1107/S1600576725009562/lan5004sup1.pdf

## Figures and Tables

**Table 1:** 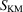
 coefficients derived by fitting the data shown in Fig. 1 ▸A with the Kubelka–Munk equation Absorption coefficients were fixed at zero since no absorber was incorporated into these materials and thaumatin crystals are transparent at 515 nm. For FAP crystals, both absorption and scattering coefficients were determined at 470 nm.

Sample	*S*_KM_ (mm^−1^)	*K*_KM_ (mm^−1^)
LCP	0.089	–
LCP + thaumatin crystals	0.204	–
HEC	0.016	–
HEC + thaumatin crystals	0.371	–
Super Lube	0.185	–
Super Lube + thaumatin crystals	2.344	–
LCP + FAP crystals (470 nm)	0.399	0.585
