# Supplementary material for: Assessing the problem of excitation light scattering in high-viscosity jets used in serial crystallography sample delivery
Source: J Appl Crystallogr. 2025 Nov 17;58(Pt 6):2078–89. doi: 10.1107/S1600576725009562 (PMC12810516; doi:10.1107/S1600576725009562)
Supplement: Supplementary file 1 [file j-58-02078-sup1.pdf]

JOURNAL OF APPLIED CRYSTALLOGRAPHY

Volume 58 (2025)

Supporting information for article:

Assessing the problem of excitation light scattering in high-viscosity jets used in serial crystallography sample delivery

Stanisław Niziński, Bogdan Marekha, Jochen Reinstein, Robert L. Shoeman, R. Bruce Doak and Ilme Schlichting

S1. Kubelka-Munk theory description

The Kubelka-Munk theory (Kubelka & Munk, 1931, Kortüm, 1969, Schuster, 1905) is based on the assumption that light incident on a material layer of defined thickness is fully diffuse and has an isotropic direction distribution. This material is characterized by a certain absorption coefficient k and scattering coefficient s. In order to reduce the complexity of the scattering phenomenon, the Kubelka-Munk model utilizes two equations, one describing collectively the light flux that travels in the forward direction and a second one that travels in the backward direction with respect to the direction normal to the material layer. One can imagine two hemispheres, one that contains all the light directions propagating in the forward direction and one in the backward direction. Therefore, the model simplifies the problem by using only one axis perpendicular to the material layer. It must be noted that since light directions are uniformly distributed, the mean light path travelled by light from x to $x+\Delta x$ will be twice as long as Δx. The derivation of this fact as well as an extensive description of scattering models in general can be found in the excellent Handbook written by Gustav Kortüm (Kortüm, 1969) that was extremely useful to us.

The Kubelka-Munk model is defined by two coupled differential equations describing forward-propagating and backward-propagating modes, respectively:

$$\begin{cases} \dfrac{dI}{dx} = -(K_{KM} + S_{KM})I + S_{KM}J \\ -\dfrac{dJ}{dx} = -(K_{KM} + S_{KM})J + S_{KM}I \end{cases} \tag{1}$$

Capital letters K_{KM} and S_{KM} denote absorption and scattering coefficients for perfectly diffuse light (as assumed in the Kubelka-Munk model). Note that $K_{KM} = 2k$ and $S_{KM} = 2s$, where lower-case letters indicate coefficients for collimated light and upper-case letters for diffuse light. S_{KM} (and s) is only associated with scattering events that send light backwards (because the forward-scattered light ends up in the same mode and cancels out in the equations). If the incident light is collimated, and the sample scattering is too low to establish isotropic light distribution in the sample, then the K_{KM} and S_{KM} coefficients determined from the Kubelka-Munk equations can be less than double of the k and s coefficients. This issue can be treated better using a more complex model that explicitly treats diffuse and collimated modes separately.

The Kubelka-Munk equations can be easily solved numerically, but analytic solutions are also available (Kortüm, 1969). The transmittance of the sample of thickness d is given by:

$$T = \frac{b}{a \sinh bS_{KM}d + b \cosh bS_{KM}d} \tag{2}$$

Where $a = (S_{KM} + K_{KM})/S_{KM}$, and $b = \sqrt{a^2 - 1}$.

S2. Integrating sphere setup

The demountable flat cells used in our experiment allow probing a relatively large flat surface of scattering material of defined thickness enclosed between two fused silica glasses, making them a suitable experimental sample enclosure system for the Kubelka-Munk model. In order to capture isotropically distributed light leaving the sample, we employed an integrating sphere (Jasco ISV-922) coupled with a Jasco V-760 UV-vis absorption spectrophotometer (see Figure S1). It allows gathering the transmitted light propagating in almost all directions (within the forward-propagating mode) and obtain a signal proportional to this light intensity using the transmission mode of operation. In this mode, a cuvette is placed at the entrance of the integrating sphere and the transmitted light is collected by the detector located at the bottom of the integrating sphere after reflecting from the sphere's internal surface. A Spectralon® White Standard Reflector is located at the reflectance port located on the opposite side of the sphere with respect to the transmittance port. A third port allows the reference light to enter the sphere, where it also reflects from the sphere's internal surface and is collected by the detector. Both "probe" and "reference" beams (Figure S1) are modulated by a chopper as in any standard UV-vis spectrometer, and collecting both of their intensities allows correction for light source fluctuations. The directly measured sample transmittance is referred to the transmittance of the reference cell. To keep the same reflection/transmission light ratio at the cuvette-sample interfaces, the reference cuvette must be filled with a clear material that has the same refractive index as the sample being studied. In our case, the same material is used for the reference measurement as for the investigated sample, but with the smallest path length available (22 μm) or by simply squeezing material between two flat glasses to get a layer as thin as possible. For samples with crystals, material without crystals will be used as a reference. This ensures an exact match of refractive indices with a negligible scattering and absorption contribution to the reference measurement. A fully transmitting sample exhibits $T=1$, while a completely non-transmitting sample has $T=0$.

The probe light incident on the sample in our experimental setup is almost fully collimated, not diffuse as assumed in the Kubelka-Munk equations. This is not necessarily a problem, provided that the isotropic light propagation direction distribution is established within the sample due to multiple scattering. If this is not the case (for example thin or weakly-scattering samples), we should observe higher than expected transmittance due to a shorter mean light path taken in the sample.

The sample of interest and the reference sample are measured consecutively in the sample port; the transmittance is calculated later. One can use the reference port for this purpose as well, *i. e.* both samples are placed consecutively in the reference port instead of the sample port and the transmittance is calculated accordingly. However, the sample port is larger than the reference port, allowing collection of a slightly larger solid angle of transmitted light (but certainly less than half of the solid angle). Therefore, we collected data using both sample and reference ports for the sake of data redundancy and error estimation. The difference between the transmittance measured using either the

reference or the sample port was used as an error. Only transmittances measured using the sample port are taken as datapoints.

Usage of the integrating sphere has clear advantages over measuring the transmittance without it, using a standard absorption spectrophotometer configuration. By using the sphere, we capture the majority of the forward-propagating diffuse mode I(x). By investigating scattering samples using the standard configuration where the spectrophotometer captures only light propagating along the probe direction, one performs a very poorly defined experiment. As one can easily observe by inserting a business card into such a spectrophotometer, the probe beam is not fully collimated, but rather astigmatic. The acceptance angle of the detection block of the spectrophotometer is not well known and depends on the specific model or brand. Therefore, using the standard geometry, one does not capture the entire forward-propagating diffuse mode I(x). One also does not capture only the directly transmitted mode (without any scattering interaction), since some fraction of the diffused light will also reach the detector. Therefore, it is unclear what the transmittances measured this way actually represent.

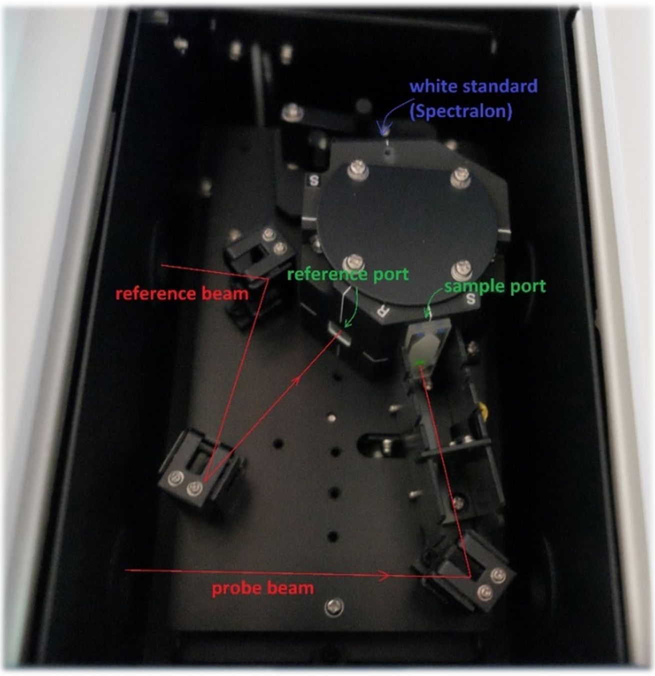

Figure S1 Jasco ISV-922 integrating sphere geometry. The internal surface of the sphere is covered with highly reflective white material, so light entering the sphere is captured by the detector regardless of its propagation angle. A Spectralon® White Standard Reflector completes the sphere by filling the port in the back of the sphere (where the sample is usually located in the reflectance mode of operation).

An interesting example (Claesson *et al.*, 2020) concerns the measurement of the transmittance of a highly scattering material using a standard UV-vis spectrometer that yielded almost zero transmittance and was interpreted as a 99% light intensity loss within the sample. However, the spectrometer did not capture the diffused light that was transmitted, but almost exclusively directly transmitted light (not scattered). This resulted in a wrong conclusion - as we demonstrate in this work - since the light that is “missing” still contributes to the overall light intensity within the sample. In fact, light rays that took the shortest path through the sample contribute less to the light intensity within the sample compared to the diffused rays. The trajectory of a diffused ray within the sample is longer and resembles a random walk. Therefore, it can effectively interact with more chromophores than directly transmitted rays that took the shortest path.

S3. Cuvette characterization

As a first approach, we use the Kubelka-Munk formalism to describe light scattering in LCP, a medium often used for viscous jets. Flat demountable fused silica cells were used, with nominal 10 µm pathlength from Starna (20-C) and 100 µm, 200 µm and 500 µm pathlength from Hellma (106-0.10-40, 106-0.20-40, 106-0.50-40). These cells consist of two glasses, one of which is completely flat and the other with a concave cavity of defined depth. Before starting the experiments, we tested whether these cells actually possess pathlengths corresponding to their nominal values. We performed two tests, first we measured the absorption spectrum of water in the 10 µm (nominal) cell with 2 nm absorption spectrometer slits. Figure S2 shows the interference pattern observed for the 10 µm cell. It is absent for cells with longer path lengths due to the wide probing bandwidth and limited coherence length of the thermal light source, therefore this approach is suitable only for the thinnest cell used. Interference maxima should obey the interference equation:

$$2\,d\,n_{H2O} = m\,\lambda_m \tag{3}$$

This assumes that the probing light is normal to the cell surface, n_{H2O} is the refractive index of water (1.33), d is the cuvette path length, λ_m is the vacuum wavelength of the interference maximum of the m-th order (meaning that the light wave transmitted through the cell without reflection constructively interferes with the doubly reflected wave within the cell cavity, and the m/2 full wavelengths fit into the cell cavity). On the spectrum plotted in the wavelength domain, the frequency of the oscillating pattern depends on the light wavelength. For two adjacent interference maxima we have:

$$T = \frac{1}{\lambda_m} - \frac{1}{\lambda_{m-1}} = \frac{m}{2\,d\,n_{H2O}} - \frac{m-1}{2\,d\,n_{H2O}} = \frac{1}{2\,d\,n_{H2O}} \tag{4}$$

Therefore, by transforming the domain of the absorption spectrum from λ to $1/\lambda$ (see Figure S2B), we obtain a constant period of the interference pattern visible on the spectrum that is simply equal to:

$$f = \frac{1}{T} = 2\,d\,n_{H2O} = 2.66\,d \tag{5}$$

The Fast Fourier transform done for water in the 10 µm specified cell with absorbance transformed to the $1/\lambda$ (frequency) domain is presented in Figure S2C; the frequency peak is located at 57.3 µm, which yields $d = \frac{f}{2.66} = 21.5$ µm true path length. This is quite unexpected for a commercial cuvette specified as 10 µm pathlength, therefore we performed another test to confirm that the path lengths of all cuvettes are well known.

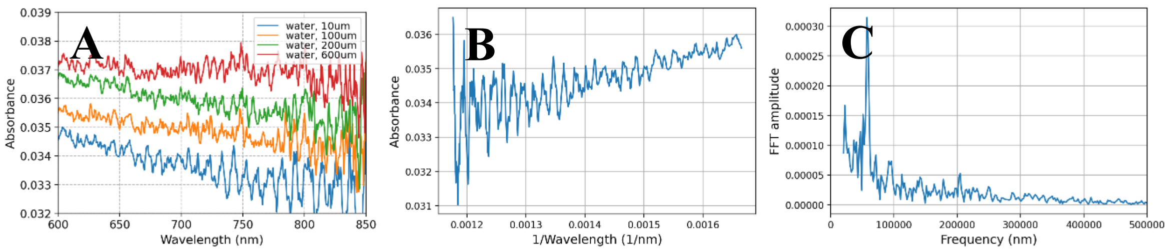

Figure S2 A) Water UV-vis absorption spectra in cuvettes of various path lengths. B) 10 µm (nominal) cell absorbance in the 600-850 nm range transformed to the $1/\lambda$ domain, C) results of the FFT.

We prepared a betanin dye solution in water (CAS-No. 7659-95-2, Sigma-Aldrich) with two concentrations and measured the absorbance at 550 nm in all cells in order to test whether they obey Beer-Lambert's law for their nominal cuvette path lengths. Figure S3B clearly demonstrates that for 100 µm, 200 µm and 500 µm cells the absorbance scales linearly with the nominal path length, while for the 10 µm cell it does not. Figure S3C shows that the absorbance scales almost linearly when one assumes that the nominally 10 µm cell has in fact 22 µm path length (as determined by the interference-based test). By assuming 24 µm path length, one obtains an even more horizontal line. Therefore, we conclude that the cuvette described as 10 µm has in fact 21.5-24 µm path length. In subsequent experiments we will use a value of 22 µm. The remaining cuvettes seem to behave in the way expected from their specifications, therefore we will assume that their nominally given path lengths are correct within a tolerance of a few µm.

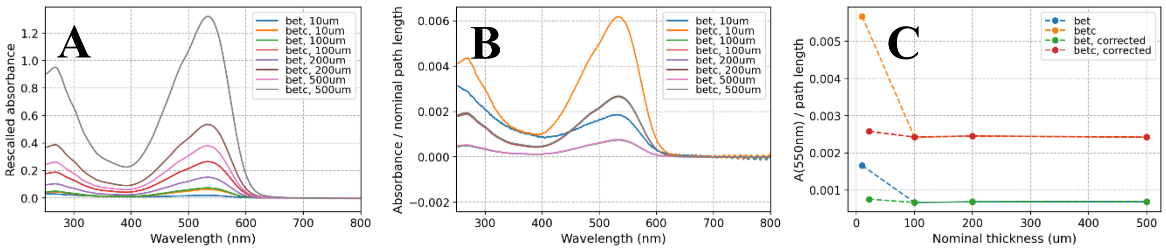

Figure S3 A) Absorbance of betanin in water with lower (bet) and higher (betc) concentration in cells of nominal path lengths of 10 µm, 100 µm, 200 µm, 500 µm. B) The same spectra divided by the nominal cell path length in µm. C) absorbance divided by cell path length for both betanin solution concentrations, before and after correcting the nominal 10 µm cell path length to 22 µm.

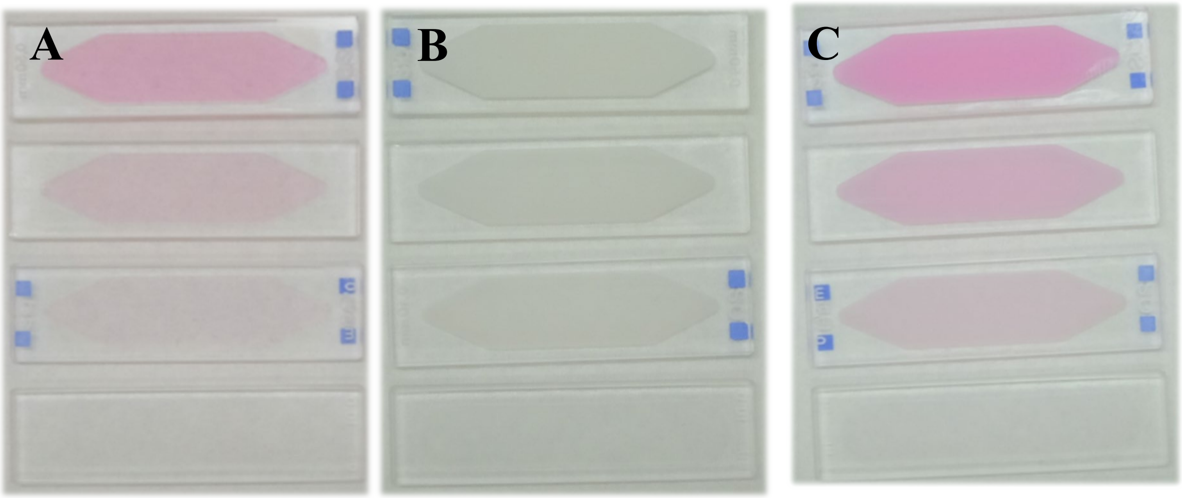

Figure S4 A) Betanin in water, B) Milk, C) Betanin in milk in cuvettes of various thicknesses (22 µm, 100 µm, 200 µm, 500 µm at the top).

S4. Testing the applicability of the Kubelka-Munk theory using milk and betanin dye

As a first step in applying the Kubelka-Munk formalism, we tested whether the model is indeed adequate to describe materials that contain inhomogeneities larger than the irradiation wavelength (such as crystals embedded in the jetting matrix) and also absorb light. To achieve this goal, we chose milk and betanin (a dye extracted from beetroot) as simple model systems that offer pure scattering and pure absorption, respectively. Betanin has a high molar absorption coefficient at ≈530 nm, negligible fluorescence quantum yield and is stable at both neutral pH and the slightly acidic pH of milk (Wendel *et al.*, 2015, Esatbeyoglu *et al.*, 2015, Strack *et al.*, 2003). Milk is a white scattering medium with no significant absorption in the visible wavelength range where betanin absorbs. Milk contains fat globules of 0.2 to 15 µm diameter (Aernouts, Van Beers, Watte, Huybrechts, Lammertyn, *et al.*, 2015b, Aernouts, Van Beers, Watte, Huybrechts, Jordens, *et al.*, 2015b, Postelmans *et al.*, 2020), which is comparable to the diameter of microcrystals used in serial pump-probe experiments. Moreover, milk has a very similar refractive index to water (≈1.35, (Jaaskelainen *et al.*, 2001)). Analysis of these samples allows to determine the absorption and scattering coefficients separately and to check whether the Kubelka-Munk model predicts the correct transmittance in a mixture of a scatterer (milk) and an absorber (betanin).

In order to determine the scattering and absorption coefficients S_{KM} and K_{KM} of a sample, it is necessary to measure the transmittance for more than one optical light path or sample thickness. Figure S5A shows the measured transmittance curves (referenced to a water layer of the same thickness) for four cuvette spacings of betanin in water (an absorbing only sample), pure milk (a scattering only sample) and betanin in milk with almost the same betanin concentration as in water. By fitting the transmittances with the Kubelka-Munk model (Figure S5A), we determined the

scattering coefficient S_{KM} and absorption coefficient K_{KM} at 515 nm for milk and betanin in water, respectively, as listed in Table S1. Purple values in the table were derived from the S_{KM} and K_{KM} values determined for scatterer only (milk) and absorber only (betanin in water), respectively. Since betanin was primarily dissolved in water, and then added to milk, the final betanin in milk sample had a diluted (by 800/840) concentration of scattering particles compared to the pure milk sample. Moreover, scattering of betanin in water is negligible, therefore probing light stays collimated and takes the shortest possible path through the sample. Since milk is highly scattering, it disorders the initially collimated probing light into diffused light, effectively doubling the mean path length of the light in the sample. These facts are taken into account when calculating the purple values in Table S1. In figure S5A, the dashed green line is not fitted, but drawn based on calculated S_{KM} and K_{KM} purple values from Table S1. Since the green dashed line overlaps well with measured transmittances (green points), we conclude that Kubelka-Munk is a suitable model to describe samples that both scatter and absorb in our measurement geometry.

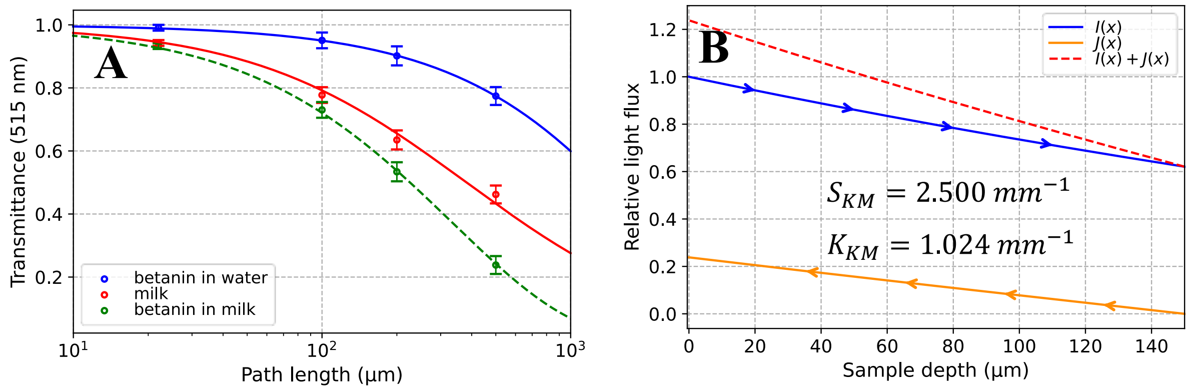

Figure S5 Analysis of the light transmittance through purely absorbing (betanin), purely scattering (milk) and both absorbing/scattering (betanin in milk) samples. A) Light transmission of the samples measured in flat cells of 22 μm, 100 μm, 200 μm and 500 μm path lengths using an integrating sphere detector. Blue and red continuous lines represent fits through the data points using the Kubelka-Munk equation. The fitting parameters are listed in Table S1. The green dashed line is based on the S_{KM} and K_{KM} parameters obtained from milk (red line) and betanin in water (blue line). B) Relative light fluxes in betanin in milk determined by numeric solution of the Kubelka-Munk equations, assuming that the incident light flux equals 1.0, the sample thickness is 150 μm and using S_{KM} = 2.500 mm^{-1}, K_{KM} = 1.024 mm^{-1} from the Table S1. I(x) is the forward-propagating light intensity, J(x) is the backward-propagating light intensity.

S5. Estimation of the diffuse mode population using the Duntley-Ryde-Silberstein model

The model developed by Duntley, Ryde and Silberstein (Ryde, 1931, Ryde & Cooper, 1931, Silberstein, 1927, Duntley, 1942, Kortüm, 1969) (we will refer to this as DRS model) can be used to extend the Kubelka-Munk formalism, by explicitly acknowledging the presence of a collimated light mode that has not yet interacted with the material - I'_x(x). Therefore, it allows to distinguish between collimated and diffuse light and estimate at which sample depth the light directionality is completely lost. It is formulated in a similar way as the Kubelka-Munk model, with an additional third equation representing collimated light:

$$\begin{cases} \frac{dI}{dx} = -(K_{DRS} + B_{DRS})I + B_{DRS}J + F'_{DRS}I'_x \\ -\frac{dJ}{dx} = -(K_{DRS} + B_{DRS})J + B_{DRS}I + B'_{DRS}I'_x \\ \frac{dI'_x}{dx} = -(K'_{DRS} + F'_{DRS} + B'_{DRS})I'_x \end{cases} \tag{6}$$

K_{DRS} indicates the absorption coefficient for the diffuse light (equivalent to K_{KM}). $I(x)$ and $J(x)$ are light intensities propagating in the forward and backward direction, respectively. These represent two half-hemispheres of the possible light directions of disordered rays present due to the scattering, as in the Kubelka-Munk model. $I'_x(x)$ is the intensity of forward propagating collimated light. At the front layer of the scattering material, $I'_x(x)$ is equal to the incident light intensity. Due to scattering interactions, this light tends to flow into diffuse modes $I(x)$ and $J(x)$. F_{DRS} and B_{DRS} are forward and backward-scattering coefficients for diffuse light, and B_{DRS} in this model is equivalent to S_{KM} in the Kubelka-Munk model. K'_{DRS}, F'_{DRS} and B'_{DRS} are the respective coefficients defined for collimated light. Due to half the mean light path along the *x* direction (light takes the shortest possible direction across material layers instead of taking random direction within full half of the solid angle), we assume that $K'_{DRS} = K_{DRS}/2$, $F'_{DRS} = F_{DRS}/2$, $B'_{DRS} = B_{DRS}/2$. These simplifications allow us to reduce this model to three parameters: K_{DRS} , B_{DRS} and F_{DRS} (listed in Table 1). The transmittance is given by the following set of formulas:

$$T = \frac{Q'bB_{DRS} - P'e^{-q'd}B_{DRS}\sinh bB_{DRS}d}{(K_{DRS} + B_{DRS})\sinh bB_{DRS}d + bB_{DRS}\cosh bB_{DRS}d} - (Q' - 1)e^{-q'd} \tag{7}$$

where:

$$Q' = \frac{(K_{DRS} + K'_{DRS})F'_{DRS} + (B_{DRS} + F'_{DRS})(B'_{DRS} + F'_{DRS})}{\left({K'_{DRS}}^2 - {K_{DRS}}^2\right) + 2K'_{DRS}(F'_{DRS} + B'_{DRS}) - 2K_{DRS}B_{DRS} + (K'_{DRS} + B'_{DRS})^2} \tag{8}$$

$$P' = \frac{(K_{DRS} - K'_{DRS})B'_{DRS} + (B_{DRS} - F'_{DRS})(B'_{DRS} + F'_{DRS})}{\left({K'_{DRS}}^2 - {K_{DRS}}^2\right) + 2K'_{DRS}(F'_{DRS} + B'_{DRS}) - K_{DRS}B_{DRS} + (K'_{DRS} + B'_{DRS})^2} \tag{9}$$

$$q' = K'_{DRS} + F'_{DRS} + B'_{DRS} \tag{10}$$

$$b = \frac{1}{B_{DRS}} \sqrt{K_{DRS}(K_{DRS} + 2B_{DRS})} \tag{11}$$

Based on this model, one can estimate how far the not-scattered, collimated beam penetrates into the sample. Figure S6A shows the same data points as plotted in Figure S5A, but fitted with the DRS model, the derived parameters are listed in Table S1. In order to obtain a good description of the transmission data points for both the milk and the betanin + milk sample, it was necessary to assume that back-scattering B_{DRS} is less than or equal to 5% of the total scattering coefficient S_{DRS}. Note that $S_{DRS} = F_{DRS} + B_{DRS}$, and B_{DRS} is an equivalent of the scattering coefficient S_{KM} determined using the Kubelka-Munk approach. Therefore, the ratio between F_{DRS} and B_{DRS} was fixed so that $F_{DRS} = 0.95$ S_{DRS} and $B_{DRS} = 0.05$ S_{DRS}. Assuming a higher back-scattering coefficient relative to the forward-scattering coefficient results in a slightly worse fit of the milk curve and a poor prediction of the milk + betanin curve. Assuming this specific ratio between F_{DRS} and B_{DRS} results in an S_{DRS} value in the order of magnitude of 50 mm^{-1}, as found in other studies (Aernouts, Van Beers, Watte, Huybrechts, Lammertyn*, et al.*, 2015a, Aernouts, Van Beers, Watte, Huybrechts, Jordens*, et al.*, 2015a, Stocker *et al.*, 2017). Based on our very limited data we cannot exclude that the contribution of forward-scattering is even higher (>95%) which would result also in a higher estimated S_{DRS} value. It is well known that light scattering by milk is highly anisotropic and that forward-scattering dominates (Aernouts, Van Beers, Watte, Huybrechts, Lammertyn*, et al.*, 2015a, Aernouts, Van Beers, Watte, Huybrechts, Jordens*, et al.*, 2015a, Postelmans *et al.*, 2020).

Figure S6B shows light intensities in a 150 µm thick sample calculated by numeric solution of the DRS equations (done using the scipy.integrate.solve_ivp function in Python) and parameters obtained from the fit of the betanin + milk data in Figure S6A. It shows that in this material the non-diffuse mode I'_x(x) is extinguished after traversing about 100 µm. In the middle (75 µm depth) the large majority of the light populates diffuse modes. Note that the total light intensity represented by the red curve in Figure S6B is almost the same as the one in Figure S5B, both models result in the same light intensity profile in the sample along the incident light direction.

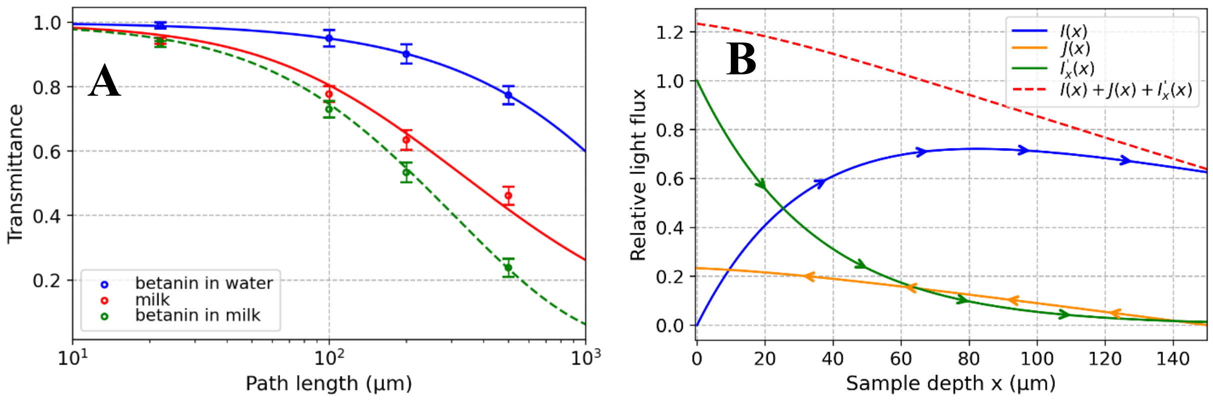

Figure S6 A) Transmittance determined with integrating sphere and associated DRS fits. B) Relative light fluxes (assuming the incident light flux equals 1.0) determined with numeric solution of the DRS equations and using S_{DRS} = 57.339 mm^{-1}, K_{DRS} = 1.024 mm^{-1} taken from Table S1 and assuming 150 μm sample thickness. *I(x)* is the forward-propagating diffuse light intensity, *J(x)* is the backward-propagating diffuse light intensity, I'_x(x) is the forward-propagating collimated light intensity.

Table S1 Obtained absorption and scattering coefficients using fit with Kubelka-Munk equation and DRS equation, respectively. For Milk without betanin, zero absorption was assumed. A doubled absorption coefficient K_{KM} for betanin in milk compared to betanin only was used due to the fact that (i) for diffuse light the mean light path is twice as long as for collimated light, and (ii) there is virtually no scattering without milk so light stays fully collimated. Note that the Kubelka-Munk equations are equivalent to Beer-Lambert's law when one assumes S_{KM} = 0. The purple font marks values that were calculated based on parameters obtained from the pure milk and betanin in water. In particular, the scattering coefficients S_{KM} and S_{DRS} for milk + betanin sample were rescaled by 800/840 with respect to pure milk due to the fact that 40 μL of the concentrated betanin solution was added to 800 μL of milk. For coefficients derived from the DRS model, the S_{DRS} = F_{DRS} + B_{DRS} dependence holds.

**Sample**	S_{KM} **(mm^{-1})**	K_{KM} **(mm^{-1})**	F_{DRS} **(mm^{-1})**	B_{DRS} **(mm^{-1})**	K_{DRS} **(mm^{-1})**
Betanin in water	-	0.512	-	-	1.024
Milk	2.625	-	57.196	3.010	-
Betanin in milk	2.500	1.024	54.472	2.867	1.024

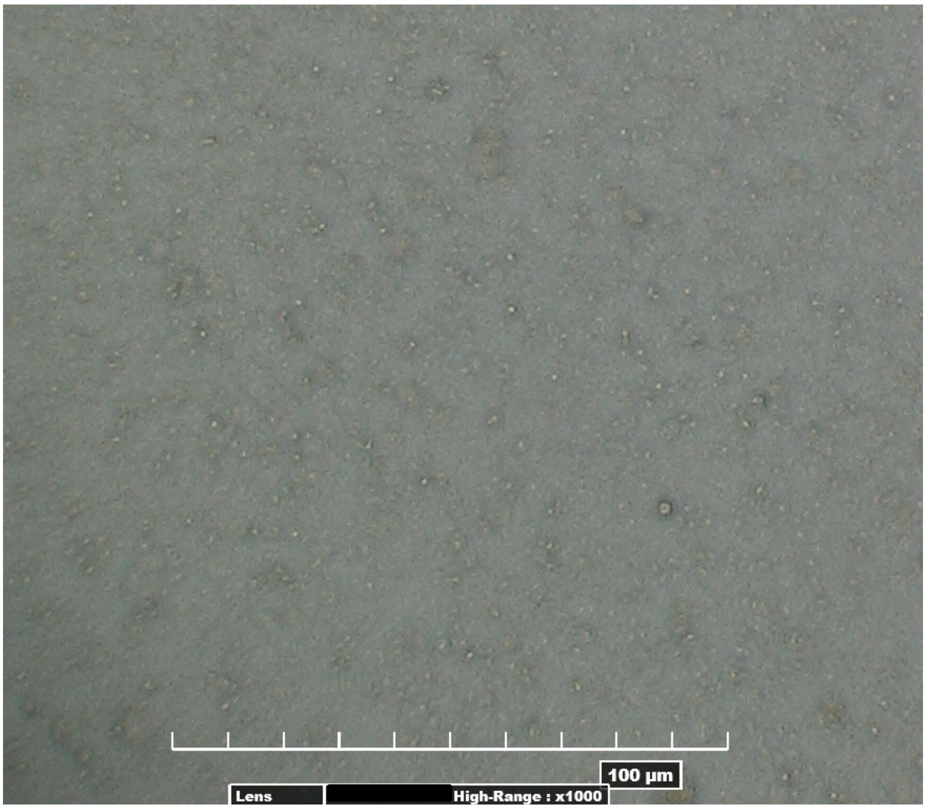

Figure S7 Optical microscopy micrograph of milk fat globules (UHT milk, 3.5 % fat). One division on the ruler represents 10 µm.

The larger the milk fat globules, the more dominant forward scattering becomes, as expected from Mie scattering theory (Aernouts, Van Beers, Watte, Huybrechts, Lammertyn*, et al.*, 2015a, Aernouts, Van Beers, Watte, Huybrechts, Jordens*, et al.*, 2015a). Because crystals commonly used in serial crystallography have dimensions comparable to or larger than milk fat globules, they are expected to have also comparable or even higher scattering anisotropy. Based on this assumption, we can apply some conclusions from the milk case to the case of protein crystals in the jetting medium. Unfortunately, neither the Kubelka-Munk nor the DRS model can be used to estimate the extent of the light contamination of the sample regions outside of the illuminated spot, since such a case violates basic assumptions used to derive them. Therefore, the usefulness of the Kubelka-Munk and DRS models is somewhat limited with respect to predicting the amount of light contamination qualitatively. Nevertheless, based on the amount of light that manages to leave the $I_x'(x)$ mode and populate the $I(x)$ and $J(x)$ modes, there are reasons to expect a certain degree of light contamination if strongly scattering materials like milk are used as jetting medium.

S6. Preparation of the jetting material samples

We found it challenging to prepare viscous samples completely free of air bubbles (see Figure S8), moreover it is hard to avoid them completely during sample mixing. We found that in our case their effect on the scattering curves is limited (the worst case is the 500 µm cuvette). Note that in samples devoid of air bubbles the transmittance will be even higher.

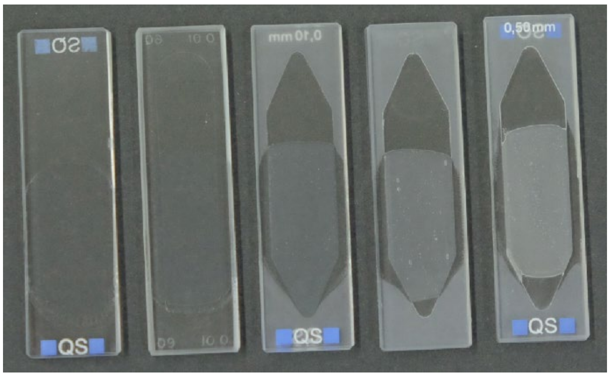

Figure S8 Exemplary set of samples (thaumatin crystals in HEC) squeezed between two flat glasses, firstly without a cavity, then with a cavity of 22 µm, 100 µm, 200 µm and 500 µm pathlengths (from left to right). Such samples were inserted into the integrating sphere, ensuring that the whole sample or reference port, respectively, was covered by the sample material.

S7. Problem of metal debris in the LCP jetting medium

One persistent issue that we frequently encounter is the presence of metal particles in the material mixed in the Hamilton syringes. The reason why in some preparations these particles are present, and in others not, is unknown. Figure S9 shows two examples of LCP preparations. Not all datapoints were collected for both samples. However, it is clear that the blue and red curves show higher scattering than preparations where no metal particles were observed.

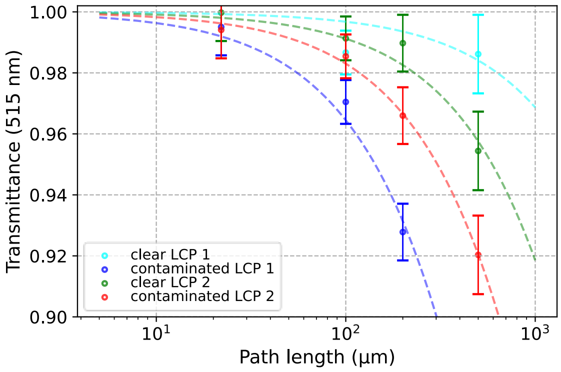

Figure S9 A) Transmittance determined with the integrating sphere and Kubelka-Munk fits for selected samples including ones with metal contamination.

We speculated that the metal debris might form when the Hamilton syringe pistons rub on the glass rim of the syringe during mixing. This idea was tested by loading the syringe that had yielded the contaminated LCP sample (blue curve) with fresh monoolein/water mixture. During this attempt the pistons were pressed vigorously more than 50 times with significant out-of-syringe-axis bending. Despite this, the resulting preparation was almost free of metal particles. Therefore, this problem is likely not caused by bent pistons/couplers/scratched syringes. When one dissolves the contaminated

sample with isopropanol and places a magnet below the waste bottle used to collect it, metal parts concentrate above the magnet after about one hour. These metal particles are also detectable by visible light microscopy (Figure S10). In the case of the contaminated LCP sample (blue curve), the metal contamination was visible even by eye. In general, all investigated LCP samples contained low, but usually negligible amounts of metal-like particles that were visible under the optical microscope.

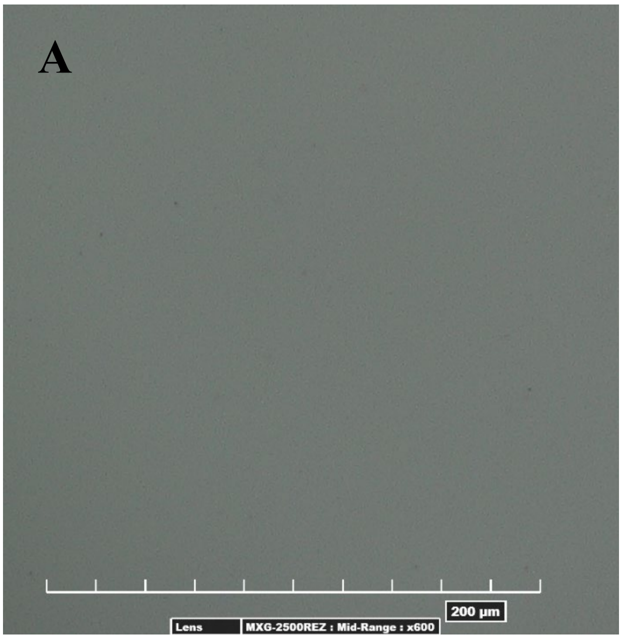

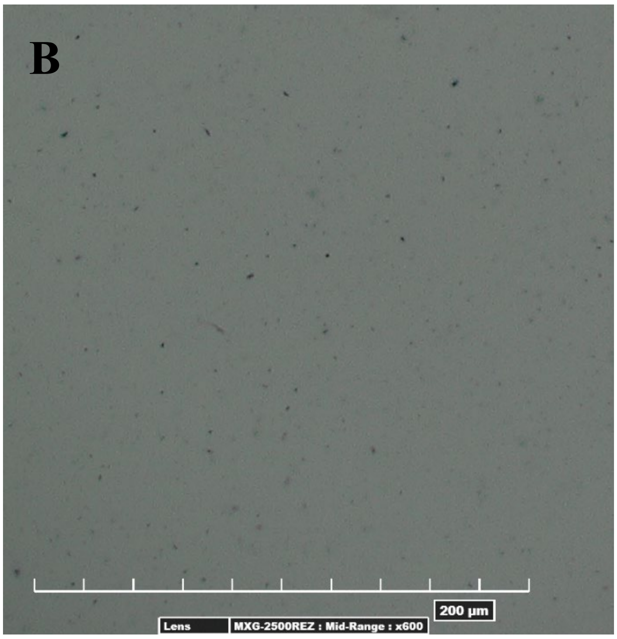

Figure S10 A) Microscope picture of the A) clear, B) contaminated LCP sample. One division on the ruler represents 20 micrometers.

S8. Estimation of the absorption coefficient for colored chromophore containing crystals of Fatty Acid Decarboxylase embedded in LCP

It is important to realize that the value of the absorption coefficient K_{KM} used in the Kubelka-Munk or K_{DRS} used in DRS formalisms is not equivalent to the absorption coefficient of a chromophore-containing crystal embedded in the jetting material. Instead, the absorption coefficient in the Kubelka-Munk equations represents the effective depletion of light in a non-homogenous medium (absorbing crystals embedded in non-absorbing medium). This effective absorption coefficient K_{KM} depends on the penetration depth of the crystal by the light, the size and concentration of crystals in the jetting medium, the chromophore concentration in the crystal and other sample and experimental geometry factors.

It is not easy to prepare crystals of the same size, refractive index and shape with and without light-absorbing chromophores (see e.g. the example in the supplement of (Nass Kovacs *et al.*, 2019)). Due

to this fact, we cannot determine K_{KM} and S_{KM} in the same way as with milk and betanin. Therefore, we used a different approach that requires two microcrystal-containing samples: LCP with thaumatin and LCP with Fatty Acid Photodecarboxylase (FAP), a flavin chromophore containing photoenzyme (Sorigue *et al.*, 2021). These samples were used as only-scattering and both scattering and absorbing sample, respectively. Thaumatin and FAP crystals do not have identical size distribution and concentration, so they scatter differently (see Figure S16 and S17). We assume that their scattering coefficients depend in exactly the same way on the light wavelength, *i. e.* $S_{FAP}(\lambda) = C_{FAP} * Q(\lambda)$ and $S_{thaum}(\lambda) = C_{thaum} * Q(\lambda)$. This is an approximation to a certain degree, as one can infer from Figure S11A. This approximation allows us to determine Q(λ) using thaumatin in LCP (since it has K_{KM} = 0 mm^{-1} in the whole spectral range registered), and to then determine S_{FAP}(470 nm) using the transmittance determined at wavelengths where FAP does not absorb light (> 550 nm). The determined scattering coefficients for thaumatin in LCP are S_{thaum}(470 nm) = 0. 236 mm^{-1} and S_{thaum}(700 nm) = 0.164 mm^{-1} (fits in Figure S11B), so Q(470 nm)/Q(700 nm) = 1.437. The determined scattering coefficient for FAP in LCP is S_{FAP}(700 nm) = 0.278 mm^{-1} (fit in Figure S11B). Therefore, we can estimate scattering coefficient for FAP in LCP at 470 nm; S_{FAP}(470 nm) = S_{FAP}(700 nm) · Q(470 nm)/Q(700 nm) = 0. 399 mm^{-1}. By fixing this value and fitting the transmission as a function of the sample path length (Figure S11B, red curve), we obtain K_{FAP}(470 nm) = 0.585 mm^{-1}. This is an exemplary value demonstrating what could be expected in real cases. It must be emphasized that this value does not characterize the material in general, but is preparation-specific, depending on the concentration and size of crystals in LCP (or other medium) and other factors.

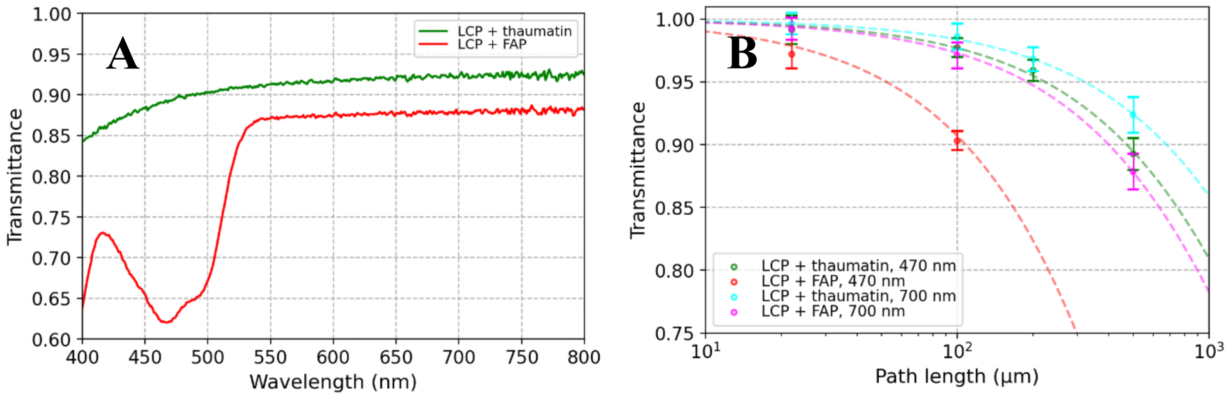

Figure S11 A) Transmittance determined for thaumatin and FAP crystals in a LCP matrix as a function of A) light wavelength (500 μm light path), B) sample light path, fitted with the Kubelka-Munk model. The absorption spectrum of the cofactor FAD is clearly visible (Sorigue *et al.*, 2021). For all curves except FAP in LCP at 470 nm, K_{KM} = 0 mm^{-1} is assumed. For FAP in LCP at 470 nm, the scattering coefficient is fixed at S_{FAP} = 0. 399 mm^{-1} and the absorption coefficient obtained from the Kubelka-Munk fit is K_{FAP} = 0.585 mm^{-1}.

S9. Effective light intensities estimated for commonly used jetting materials

Figure S12A shows the measured transmissions of various jetting materials with and without colourless thaumatin crystals embedded (microscope pictures in Figure S14, S15 and S16) fitted with the Kubelka-Munk model. Table S2 shows that both HEC and LCP exhibit low scattering coefficients at 515 nm. As shown in Figure S12B, the scattering increases for shorter wavelengths. The addition of thaumatin crystals to HEC and LCP media results in a significant scattering increase (0.016 mm^{-1} → 0.371 mm^{-1} and 0.089 mm^{-1} → 0.204 mm^{-1}, respectively). Still, the only material scattering comparably to the previously discussed milk sample is Super Lube™ grease with thaumatin crystals.

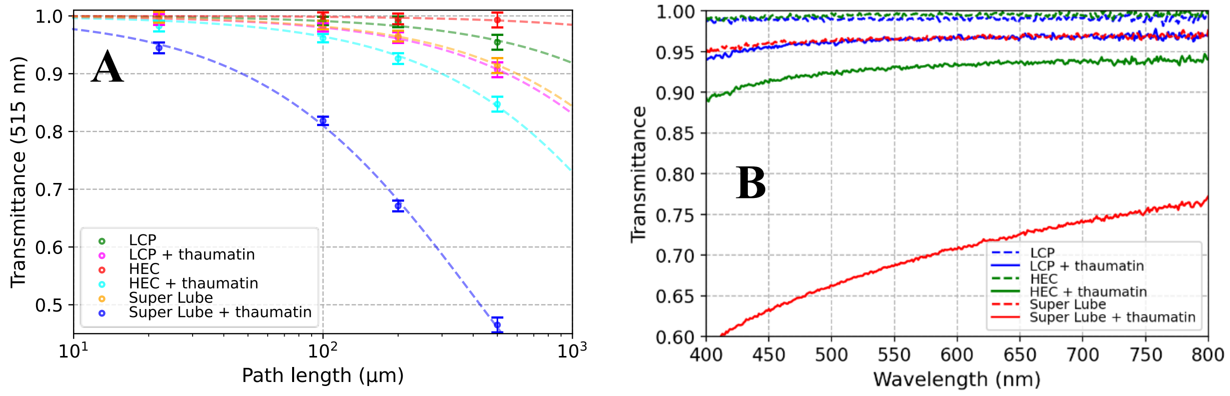

Figure S12 A) Transmittance determined with an integrating sphere and Kubelka-Munk fits. B) Transmittance plotted as a function of light wavelength (200 µm path length).

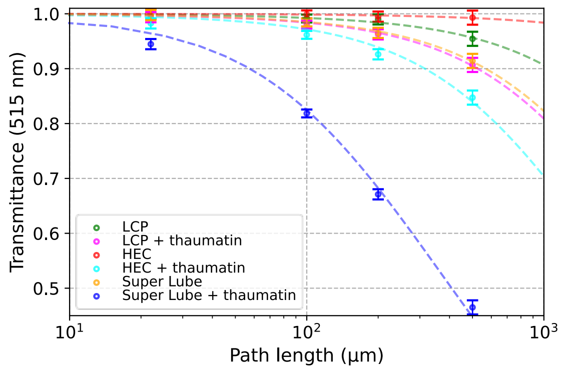

Figure S13 A) Transmittance determined with an integrating sphere and DRS fits. The backward-to-forward scattering ratio was arbitrarily fixed: B_{DRS} = 0.05 S_{DRS}, F_{DRS} = 0.95 S_{DRS}. A slightly better fit of the 22 µm datapoint can be obtained by assuming even more pronounced forward scattering, however we prefer to explicitly fix this value since it cannot be accurately estimated using our data.

Table S2 S_{KM} coefficients obtained using fits with the Kubelka-Munk equation and F_{DRS} and B_{DRS} coefficients using the DRS model. Absorption coefficients were fixed at zero since no absorber was incorporated in these materials and thaumatin crystals are transparent at 515 nm. For FAP crystals in LCP, both coefficients were derived for 470 nm.

**Sample**	S_{KM} (mm^{-1})	K_{KM} (mm^{-1})	F_{DRS} (mm^{-1})	B_{DRS} (mm^{-1})	K_{DRS} (mm^{-1})
LCP	0.089	-	2.760	0.145	-
LCP + thaumatin	0.204	-	5.604	0.295	-
HEC	0.016	-	0.559	0.029	-
HEC + thaumatin	0.371	-	9.340	0.492	-
Super Lube™	0.185	-	5.177	0.272	-
Super Lube™ + thaumatin	2.344	-	51.159	2.693	-
LCP + FAP	0. 399	0.585	-	-	-

Milk has fat globules of approximately a few µm diameter (see Figure S7). This results in a strong forward-scattering and a minuscule back-scattering coefficient which represents less than 5% of the total scattering. The thaumatin crystals have diameters in the 10-20 µm range (Figures S14, S15, S16), *i. e.* a few times larger than the fat globules in milk (note that they are more likely to orient their long axis parallel to the microscope glass slide, so their dimension along the microscope axis is presumably smaller). This should result in higher scattering anisotropy, where forward scattering is even more favoured (Postelmans *et al.*, 2020, Aernouts, Van Beers, Watte, Huybrechts, Jordens*, et al.*, 2015a, Aernouts, Van Beers, Watte, Huybrechts, Lammertyn*, et al.*, 2015a). Since we cannot easily estimate the ratio of forward- to backward-scattering, we assume F_{DRS} = 0.95 S_{DRS} as in the milk case.

We simulated the light intensities of the forward-propagating mode *I(x)* and the backward-propagating mode *J(x)* in a 150 µm thick sample (Figure S18) using the Kubelka-Munk approach and the scattering coefficients listed in Table S2. We examined thaumatin crystals in HEC (S_{KM} = 0.371 mm^{-1}) and thaumatin crystals in Super Lube™ (S_{KM} = 2.344 mm^{-1}) as examples of low and high scattering samples. For the low-scattering case, the addition of a K_{KM} = 1 mm^{-1} absorber results in a total light intensity of 93% of the incident light at 75 µm sample depth (Figure S18C), ~0.2% less than for the case with S_{KM} = 0 mm^{-1} and K_{KM} = 1 mm^{-1} (not shown). This demonstrates that thaumatin crystals in both HEC and LCP experience almost the same light intensity as in the case without any scattering. The high-scattering case is different, although the light intensity decay of the forward-propagating mode is compensated mostly by the back-propagating mode. With the absorption coefficient of K_{KM} = 1 mm^{-1}, the total light intensity fraction of the incident light at 75 µm sample

depth is 92% (Figure S18D). This is almost the same value as in the low-scattering case. This shows that scattering does not reduce the average light intensity impinging on the crystals, it only disorders the light flow in the sample. However, this could be a potential problem for very high temporal resolution experiments if a significant fraction (for example 20%) of the incident light intensity travels in the backwards mode close to the front layer of the sample (like in Figure S18D). Simplifying, this is comparable to a situation where the pump pulse is reflected back in the middle of the sample (75 µm position). Therefore, for a given pump-probe delay *t* and a crystal located at the front surface of the sample (0 µm position) the recorded scattering pattern will have a ~20% contribution from a *t* - 0.75 ps pump-probe delay (corresponding to the additional 150 µm optical path travelled by the back-scattered pulse in n=1.5 medium). Estimating the exact magnitude of this issue is beyond the scope of this work, but in the discussed geometry one can expect non-negligible "smearing out" of the temporal resolution on a magnitude of ~0.75 ps.

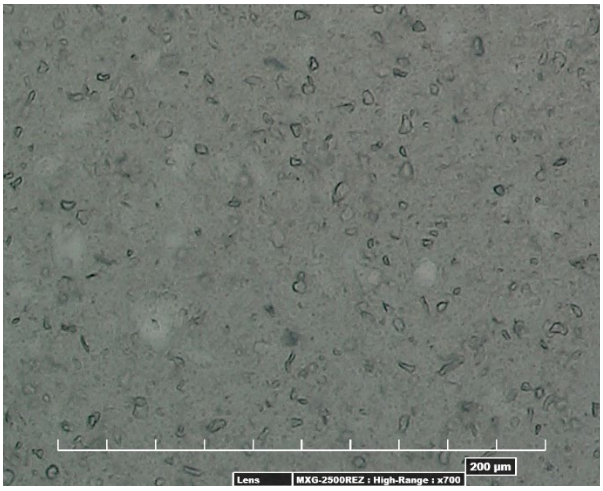

Figure S14 Thaumatin crystals in Super Lube™.

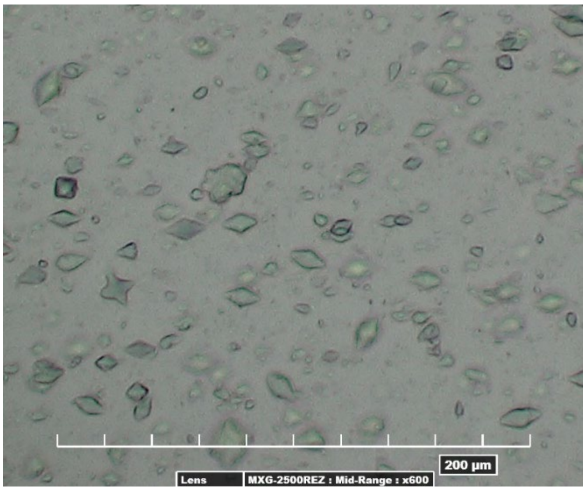

Figure S15 Thaumatin crystals in HEC.

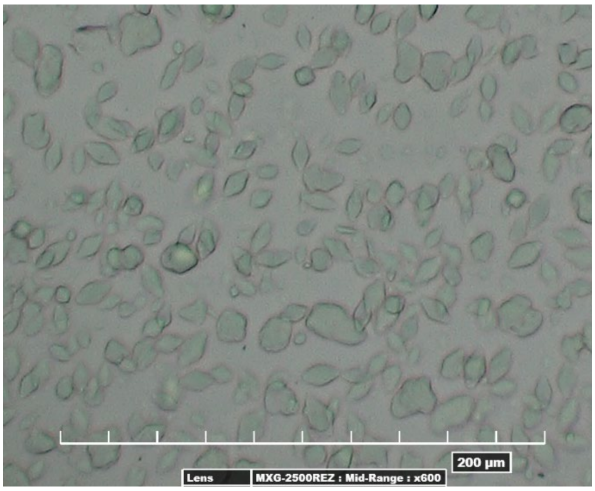

Figure S16 Thaumatin crystals in LCP.

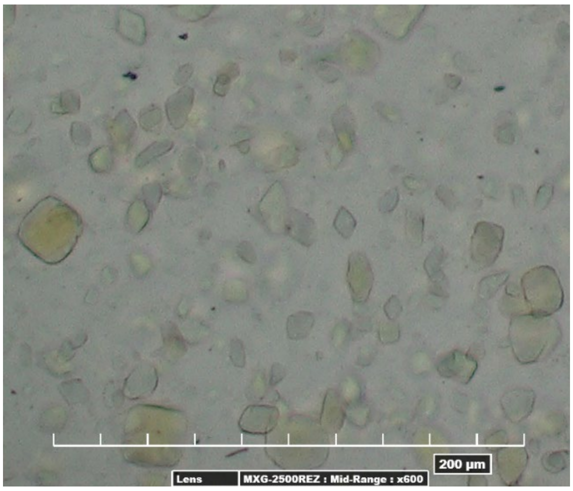

Figure S17 FAP crystals in LCP.

Note that one division on the ruler represents 20 micrometers for Figures S14-S17.

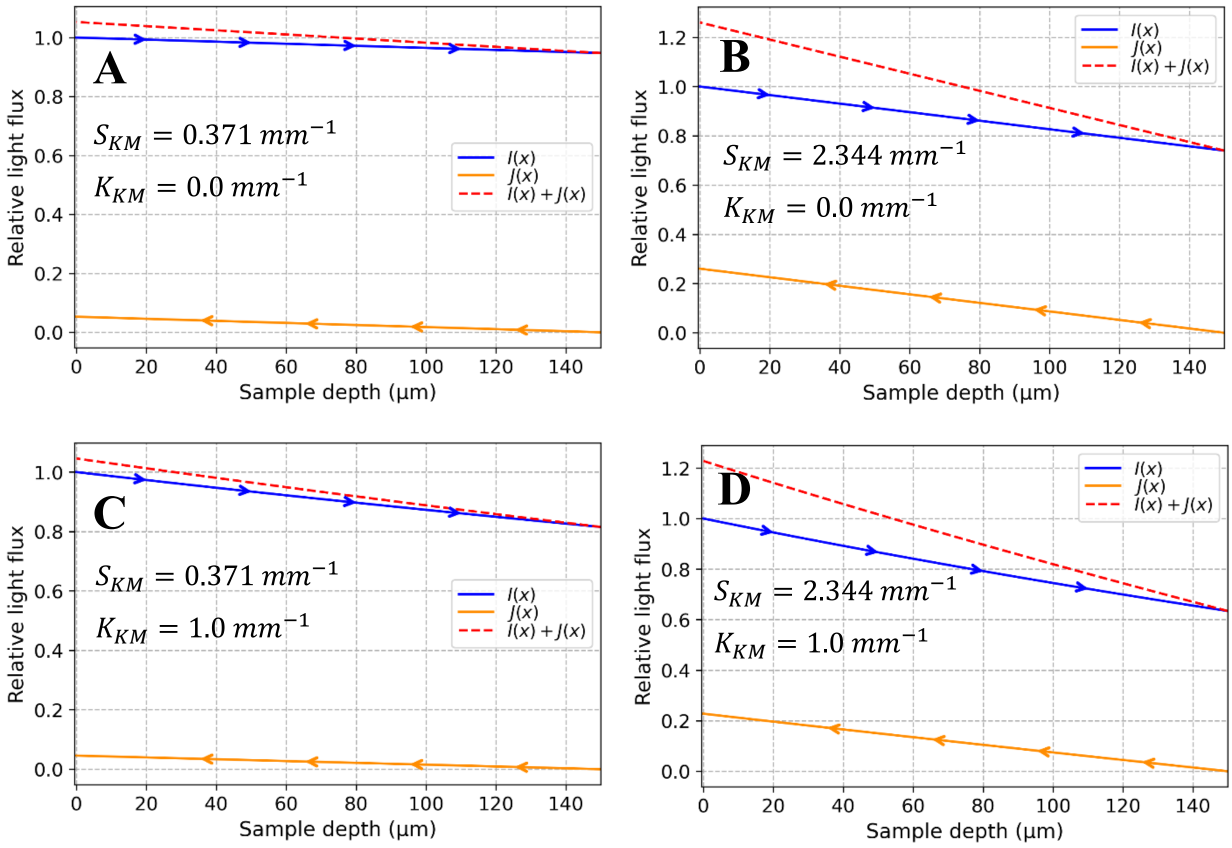

Figure S18 Light intensity estimated from the numerical solution of the Kubelka-Munk equations. A) and C) describe the low-scattering case of thaumatin in HEC (S_{KM} = 0.371 mm^{-1}) while B) and D) describe the highly-scattering case of thaumatin in Super Lube™ (S_{KM} = 2.344 mm^{-1}). For A) and B) no absorber is assumed (K_{KM} = 0 mm^{-1}), for C) and D) K_{KM} = 1 mm^{-1} is assumed.

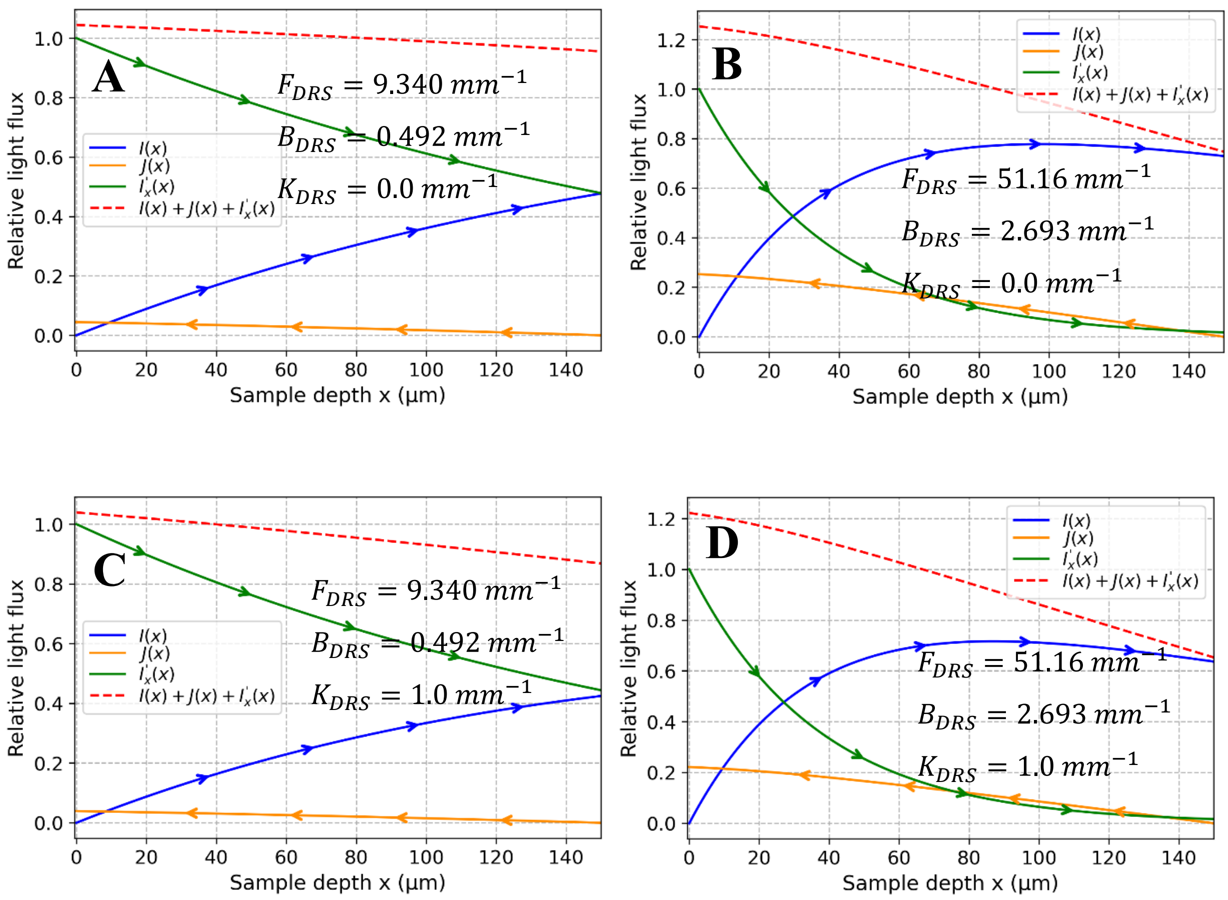

Figure S19 Light intensity estimated from the DRS numerical solution. A) and C) describe the low-scattering case of thaumatin in HEC (F_{DRS} = 9.340 mm^{-1} and B_{DRS} = 0.492 mm^{-1}) while B) and D) describe the highly-scattering case of thaumatin in Super Lube™ (F_{DRS} = 51.159 mm^{-1} and B_{DRS} = 2.693 mm^{-1}). For A) and B) no absorber is assumed (K_{DRS} = 0 mm^{-1}), for C) and D) K_{DRS} = 1 mm^{-1} is assumed.

The simulations using the DRS model result in a similar total light intensity profile across the sample depth as for the Kubelka-Munk approach. These results, although much more speculative than the Kubelka-Munk derived ones, show that for thaumatin crystals in HEC about half of the non-absorbed light intensity will migrate to diffuse modes (see Figure S19C), resulting in limited potential for light contamination of a sample volume that was not directly irradiated. Still, the back-propagating diffuse mode *J(x)* contains almost negligible intensity here, and one can expect that most of the scattered rays interacted only once, and still travel predominantly in the forward direction, displacing light from the area of focus only to a limited extent. By contrast, the case of thaumatin crystals in Super Lube™ is comparable to milk, the collimated mode I'_x(x) decays entirely within a 150 µm thick sample (Figure S19D). Regardless of the added absorption coefficient, all light ends up in diffuse modes, indicating a

huge potential to light-contaminate a sample volume that has not been directly irradiated. This agrees well with the visual observation that it is impossible to see objects through Super Lube™ + thaumatin crystals samples of thickness > 100 µm (see Figure S20) in contrast to that for the HEC and LCP samples with thaumatin crystals (Figure S21).

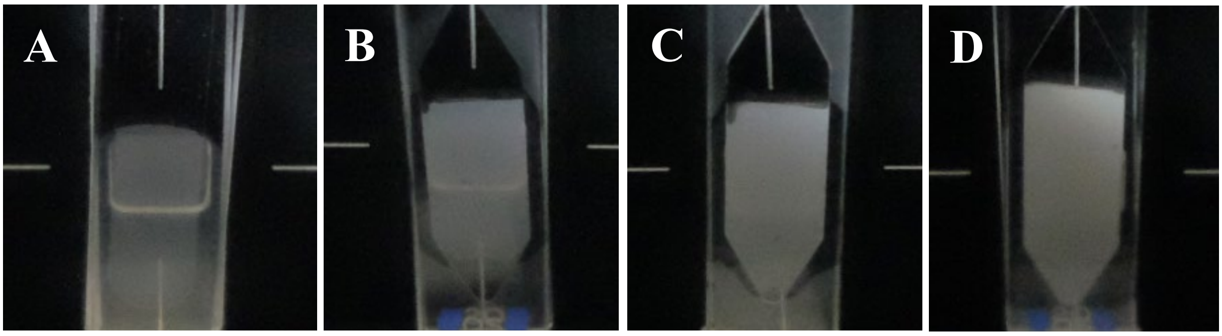

Figure S20 Thaumatin crystals in Super Lube™ in A) 22 µm, B) 100 µm, C) 200 µm, D) 500 µm cuvette path photographed at the reference port of the integrating sphere. The bottom edge of the integrating sphere port is barely visible through the sample for 22 µm and 100 µm path lengths, but it is no longer visible for ≥200 µm path length. This confirms that the collimated mode $I_x'(\mathrm{x})$ is fully extinguished between 100 µm and 200 µm path length, in accordance with the DRS model description. Note that light can propagate effectively further than that, but only within the diffuse mode, so the information required to generate the image is lost.

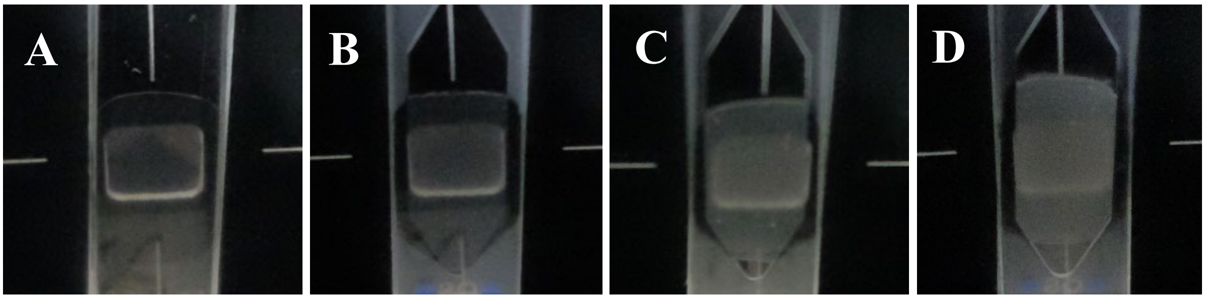

Figure S21 Thaumatin crystals in HEC in A) 22 µm, B) 100 µm, C) 200 µm, D) 500 µm cuvette path photographed at the reference port of the integrating sphere. The bottom edge of the integrating sphere port is visible for all path lengths, confirming that even for 500 µm path length the $I_x'(\mathrm{x})$ mode persists, in accordance with the DRS model.

The Kubelka-Munk and DRS models are both huge simplifications and do not allow derivation of the light intensity profile for directions perpendicular to the light propagation. However, the models are sufficient to show that light scattering does not only lead to a decrease of the light intensity in the back layers of the sample, but also to an increase in the sample layers close to the front surface. The presence of the back-propagating mode also means that due to scattering, some regions of the crystal can potentially experience light exposure that would not have occurred if the light had propagated only in the forward direction. In the case of large and highly absorbing protein crystals, where light cannot penetrate through the crystal, it can counter-intuitively lead to higher occupancy, since a back-propagating mode can illuminate it from the dark side. One has to also mention that our considerations assume a homogenous sample, while in a 150 µm jet there may be a very limited, stochastically changing number of crystals within the illuminated region. This will obviously result in shot-to-shot variations in the pump light intensity that crystals experience at the X-ray focus (which is typically much smaller than the pump) during data collection.

S10. Why do thaumatin crystals prepared in Super Lube™ show dramatically increased scattering compared to pure Super Lube™ and thaumatin crystals in LCP?

We tested whether the strong increase in scattering by Super Lube™ (S_{KM} = 0.185 mm^{-1} → 2.344 mm^{-1}) after adding the crystal slurry was due to added crystals or added water. To this end, we evaluated the scattering of pure water mixed with Super Lube™. Since Super Lube™ (or any PTFE grease) is highly hydrophobic, we expected that due to lack of mixing between water and grease, tiny water bubbles can form (but larger than visible light wavelength) and that these lead to increased scattering. Figure S22 shows the effects of adding water to Super Lube™. At about 2-4% water content, the sample becomes very cloudy, and after about 10% water content the sample scattering is so high that it is impossible to see the PTFE particles under the microscope. This effect presumably resulted in the poor visibility of the thaumatin crystals in Super Lube™ as well. We expect that the same problem will affect any hydrophobic jetting material, and it may be impossible to completely avoid adding water to the grease together with the crystals. This is another point in favor of HEC and LCP matrices instead of Super Lube™ (or hydrophobic matrices in general).

Figure S23A demonstrates the transmittance of Super Lube™ with 10% water content (yielding the S_{KM} of about 5.9 mm^{-1}) compared to Super Lube™ (S_{KM} = 0.185 mm^{-1}, see Table S2) and Super Lube™ with thaumatin crystals (S_{KM} = 2.344 mm^{-1}, see Table S2). This result demonstrates that the water content present in the thaumatin crystal slurry is sufficient to increase the scattering of the mixed sample by an order of magnitude, while thaumatin crystals presumably contribute less to the scattering increase. Strongly scattering sample makes it also harder to see the embedded crystals under the microscope. Note that the thaumatin crystal slurry was obtained by centrifugation, removing the supernatant completely. A small amount of mother liquor was added to make the slurry “just”

pipettable. Hence, it would be extremely difficult, if not impossible to reduce the amount of liquid introduced into the Super Lube™ matrix.

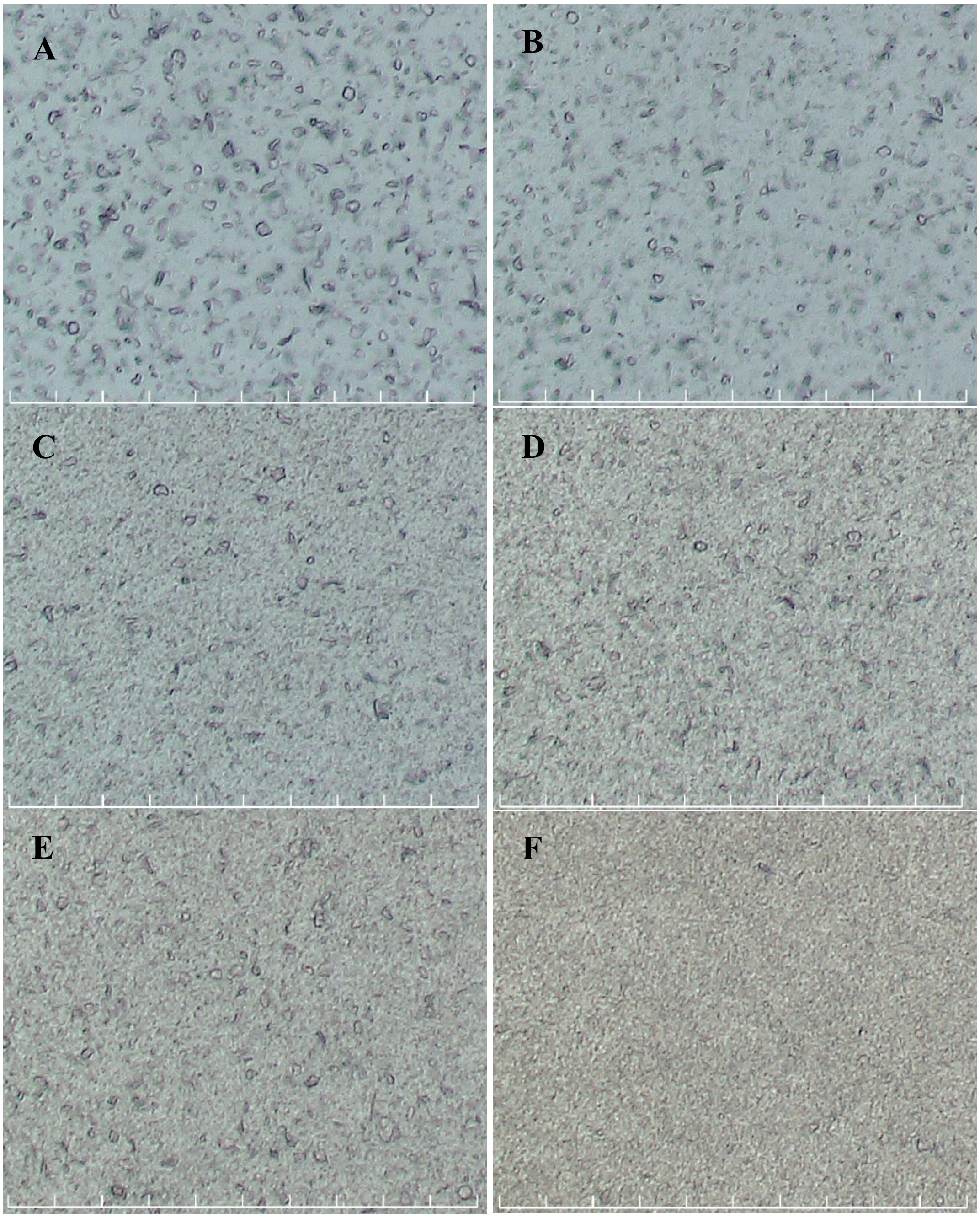

Figure S22 Super Lube™ with various amounts of water added (the horizontal scale indicates 200 μm in total). The water content is approx. A) 0%, B) 1%, C) 3%, D) 5%, E) 8%, F) 10%. For the B-E cases, inaccuracy of adding the water was very high (since single microliters of water have been added incrementally into a Hamilton syringe for syringe mixing with 90 μl Super Lube™ in the other syringe), therefore these values should be treated as very approximate.

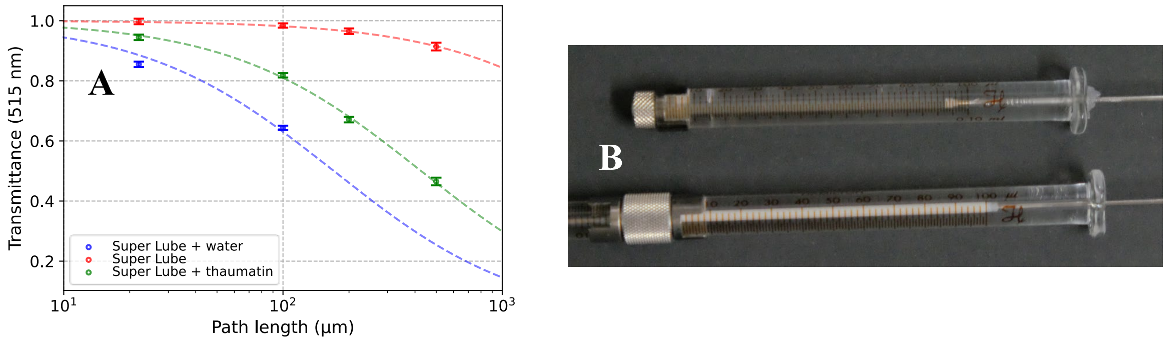

Figure S23 A) Transmittances measured for pure Super Lube™, Super Lube™ with thaumatin crystals (and certain amount of water) and Super Lube™ mixed with water (10%). B) Upon addition of 10% water, pure Super Lube™ (upper syringe) becomes completely white due to strong scattering (lower syringe).

S11. Transient absorption experiment with displaced pump beam versus probe beam

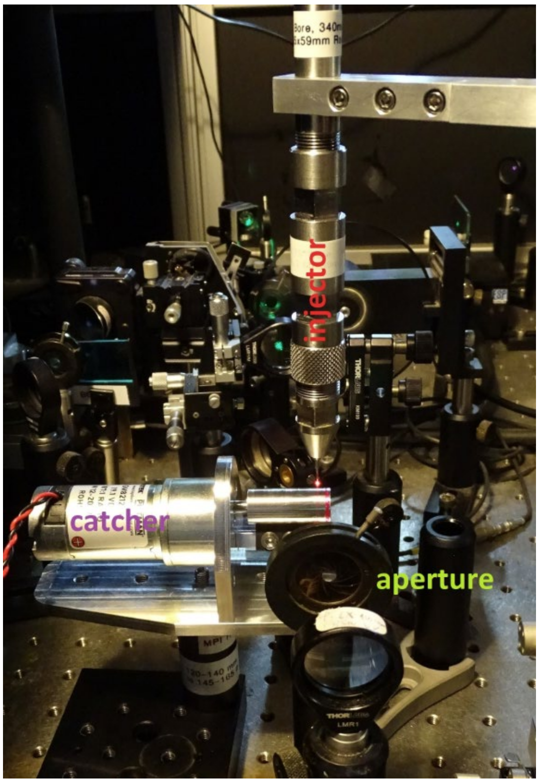

Figure S24 Setup of the time-resolved absorption spectroscopy experiments on actual running viscous jets. The jetting sample is delivered from the top using a high viscosity extruder (labeled injector). The fluorescence of Nile Red contained in the jetting material is visible in the interaction zone above the rotary catcher. It serves as an attachment for the jet, thereby stabilizing it and collecting the spent material (Doak *et al.*, 2023). Pump and probe beams travel through the vertical jet axis. A downstream aperture in front of the catcher rejects the pump beam, while the probe beam travels through the collimating lens (downstream of the aperture). The pump was displaced vertically along the jet axis by moving the focusing lens behind (upstream of) the high viscosity extruder.

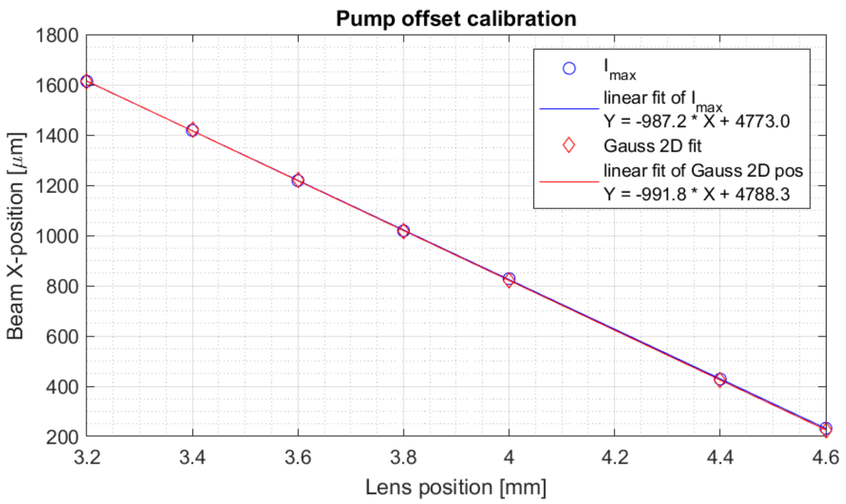

Figure S25 Focus position along the jet determined using a beam profiler as a function of the lens position determined using two different methods. In one method, the maximum intensity point registered by the beam profiler was taken, in the other it was fitted by a 2D Gaussian function and its peak position was determined. Both methods yield very similar calibration curves.

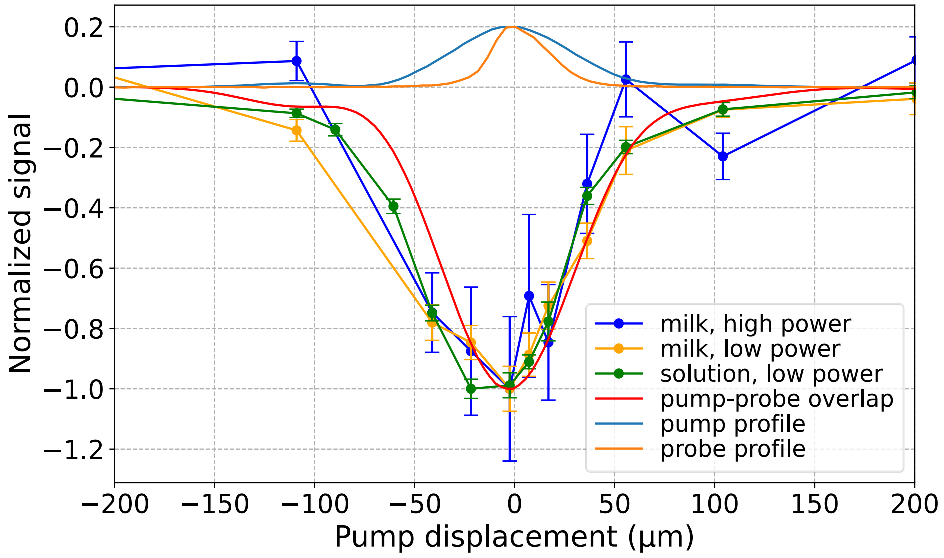

Figure S26 Preliminary transient absorption experiment on Rhodamine B in water and in milk with the pump beam spatially offset by a varying distance from the probe position. The signal was obtained at a pump probe delay of 30 ps, and averaged in the 560-610 nm range. The 'low power' data correspond to an excitation peak energy density of ~0.66 mJ/cm^2, which is far below the saturation level of Rhodamine B (~2 mJ/cm^2). To check, if upon saturation of the absorption, a larger fraction of the pump photons will be scattered and will result in some broadening we also collected 'high power' data (excitation peak energy density ~4.1 mJ/cm^2). No broadening was observed, although the raw data are noisier due to stronger overall scattering. If the scattered light propagated beyond the laser spot dimensions, a transient absorption signal would be observed even when the laser beams no longer overlap.

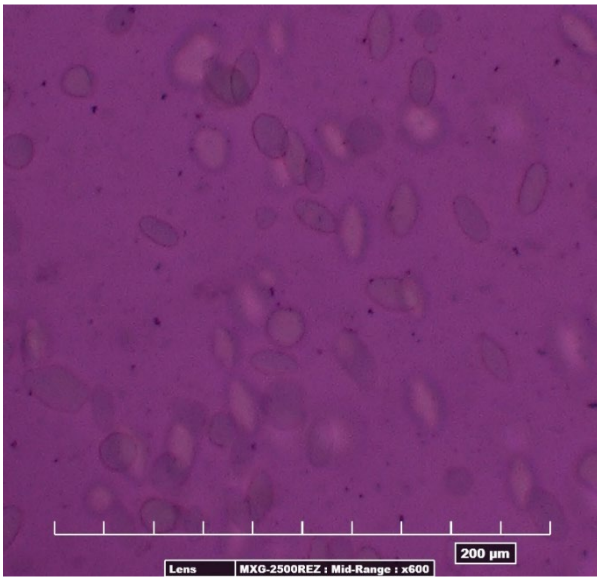

Figure S27 Thaumatin crystals in LCP with Nile Red dye used in transient absorption spectroscopy experiment.

S12. Detailed description of Monte-Carlo simulations in jet geometry using MCCL package

Figure S28 shows the fluence cross-section cutting across the jet (infinite cylinder) for three cases, demonstrating that moderate light scattering (case B, comparable to thaumatin crystals in HEC or LCP) counteracts jet lensing effects, but also results in a certain degree of light redistribution within the irradiated zone, making it more uniform compared to the non-scattering case (A). In Figure S28C, high scattering (comparable to thaumatin crystals in Super Lube™) results in very low beam penetration through the cylinder and an increased light fluence at its front surface. Figure S30 shows how the fluence profiled along axes parallel to the infinite cylinder (Y-axis) changes before entering, within and after leaving the cylinder. For μ_s=9.8 mm^{-1} case (B), all profiles are very similar, demonstrating that moderate scattering is beneficial since it counteracts the jet lensing effect and leads to a more uniform light fluence profile within the jet. The high scattering case (μ_s=53.8 mm^{-1}) results in extended tails of the fluence distribution (*i.e.,* light contamination), and light trapping within the jet, resulting in decreased transmission (Figure S30C, red curve). Figure S31 shows how the jet lensing effect distorts the fluence profile along the X-axis, which is perpendicular to both the beam direction and the cylinder axis.

This demonstrates that even under strong scattering conditions (case C), the average fluence decrease is very limited in the jet slice irradiated directly by the laser. In fact, moderate levels of scattering (such as for thaumatin crystals in HEC or LCP) could be beneficial in jet sample delivery, since they smooth the light intensity profile within the interaction region. Stronger scattering (case C) leads to light contamination effects and prevents the laser beam from fully penetrating the jet, while increasing

the light intensity in the frontal volume of the jet (where the laser beam impinges), leading again to a non-uniform light intensity profile. In conclusion, the mean light intensity within the irradiated jet section does not depend strongly on the scattering magnitude, however, the uniformity of the light intensity within this section does.

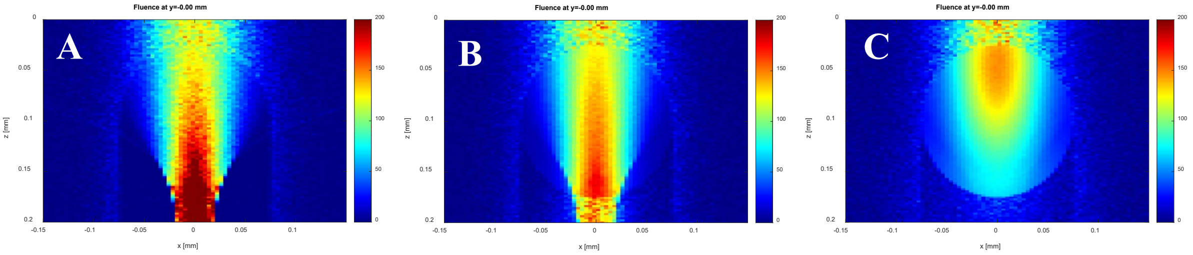

Figure S28 Simulated fluence profiled along the beam propagation direction (along Z) and perpendicular to the infinite cylinder axis (along Y). It is plotted at Y=0 mm, which represents the centre of the light beam cross-section profile. Scattering coefficients are A) μ_s=0.1 mm^{-1}, B) μ_s=9.8 mm^{-1}, C) μ_s=53.8 mm^{-1}.

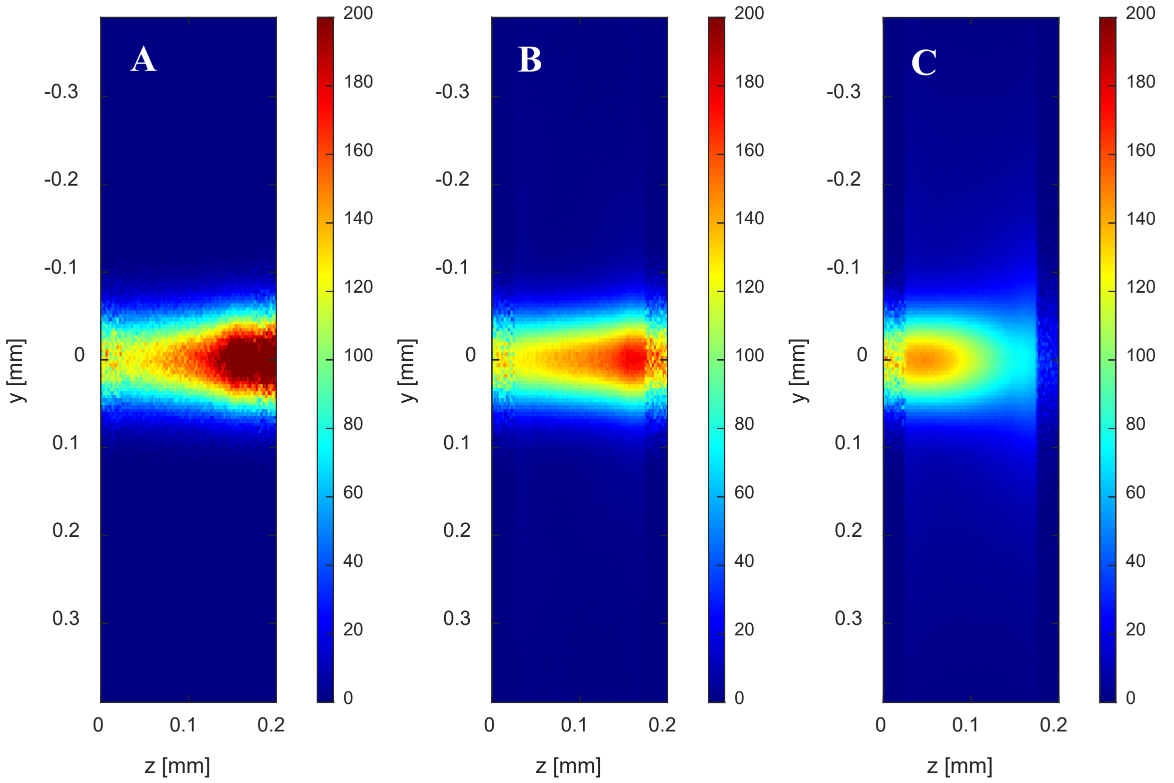

Figure S29 Simulated fluence profile on the YZ plane, at x=0 (slicing through the centre of the beam and the jet). Scattering coefficients are A) μ_s=0.1 mm^{-1}, B) μ_s=9.8 mm^{-1}, C) μ_s=53.8 mm^{-1}. For the case C, cylinder (jet) edges are visible due to light propagating upstream and downstream from the irradiated region.

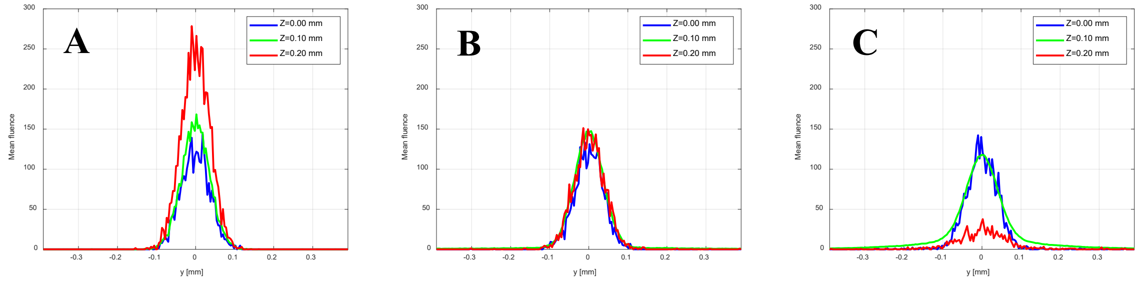

Figure S30 Simulated fluence profile along the Y axis (the cylinder axis), at x=0 (centre of the beam and the jet) plotted before the jet (z=0 mm), within the jet (z=0.1 mm) and after the jet (z=0.2 mm). Scattering coefficients are A) μ_s=0.1 mm^{-1}, B) μ_s=9.8 mm^{-1}, C) μ_s=53.8 mm^{-1}. The green curve in case C shows the extent of light contamination possible in strong scattering case.

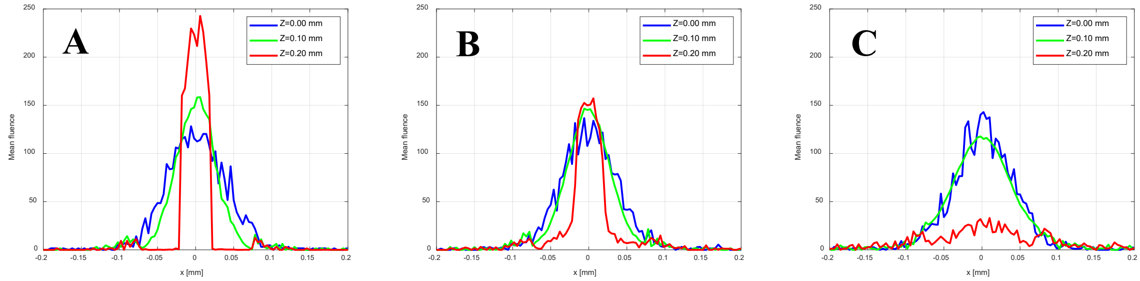

Figure S31 Simulated fluence profile along the X axis, at y=0 (centre of the beam) plotted before the jet (z=0 mm), within the jet (z=0.1 mm) and after the jet (z=0.2 mm). Scattering coefficients are A) μ_s=0.1 mm^{-1}, B) μ_s=9.8 mm^{-1}, C) μ_s=53.8 mm^{-1}. Due to the focusing effect, the red curve is much narrower and sharper than initial (blue) beam cross-section profile. Note that in case B all three profiles have comparable peak height, because scattering redirects the light effectively from the focal point just enough to compensate for the lensing effect.

References

Aernouts, B., Van Beers, R., Watte, R., Huybrechts, T., Jordens, J., Vermeulen, D., Van Gerven, T., Lammertyn, J. & Saeys, W. (2015a). *Colloids Surf B Biointerfaces* **126**, 510-519.

Aernouts, B., Van Beers, R., Watte, R., Huybrechts, T., Jordens, J., Vermeulen, D., Van Gerven, T., Lammertyn, J. & Saeys, W. (2015b). *Colloids Surf B Biointerfaces* **126**, 510-519.

Aernouts, B., Van Beers, R., Watte, R., Huybrechts, T., Lammertyn, J. & Saeys, W. (2015a). *J Dairy Sci* **98**, 6727-6738.

Aernouts, B., Van Beers, R., Watte, R., Huybrechts, T., Lammertyn, J. & Saeys, W. (2015b). *J Dairy Sci* **98**, 6727-6738.

Claesson, E., Wahlgren, W. Y., Takala, H., Pandey, S., Castillon, L., Kuznetsova, V., Henry, L., Panman, M., Carrillo, M., Kübel, J., Nanekar, R., Isaksson, L., Nimmrich, A., Cellini, A., Morozov, D., Maj, M., Kurttila, M., Bosman, R., Nango, E., Tanaka, R., Tanaka, T., Fangjia, L., Iwata, S., Owada, S., Moffat, K., Groenhof, G., Stojković, E. A., Ihalainen, J. A., Schmidt, M. & Westenhoff, S. (2020). *eLife* **9**, e53514.

Doak, R. B., Shoeman, R. L., Gorel, A., Barends, T. R. M., Marekha, B., Haacke, S., Nizinski, S. & Schlichting, I. (2023). *J. Appl. Crystallogr.* **56**, 903-907.

Duntley, S. Q. (1942). *J. Opt. Soc. Am.* **32**, 61-70.

Esatbeyoglu, T., Wagner, A. E., Schini-Kerth, V. B. & Rimbach, G. (2015). *Mol Nutr Food Res* **59**, 36-47.

Jaaskelainen, A. J., Peiponen, K. E. & Raty, J. A. (2001). *J Dairy Sci* **84**, 38-43.

Kortüm, G. (1969). *Reflectance spectroscopy Principles, methods, applications*. New York: Springer-Verlag.

Kubelka, P. & Munk, F. (1931). *Z. Tech. Physik* **12**, 593–601.

Nass Kovacs, G., Colletier, J. P., Grunbein, M. L., Yang, Y., Stensitzki, T., Batyuk, A., Carbajo, S., Doak, R. B., Ehrenberg, D., Foucar, L., Gasper, R., Gorel, A., Hilpert, M., Kloos, M., Koglin, J. E., Reinstein, J., Roome, C. M., Schlesinger, R., Seaberg, M., Shoeman, O. L., Stricker, M., Boutet, S., Haacke, S., Heberle, J., Heyne, K., Domratcheva, T., Barends, T. R. M. & Schlichting, I. (2019). *Nat. Commun.* **10**, 3177.

Postelmans, A., Aernouts, B., Jordens, J., Van Gerven, T. & Saeys, W. (2020). *Innov Food Sci Emerg* **60**.

Ryde, J. W. (1931). *Proc. Roy. Soc. London A* **131**, 451-464.

Ryde, J. W. & Cooper, B. S. (1931). *Proc. Roy. Soc. London A* **131**, 464-475.

Schuster, A. (1905). *Astrophys J* **21**, 1-22.

Silberstein, L. (1927). *Phil. Mag.* **4**, 1291-1296.

Sorigue, D., Hadjidemetriou, K., Blangy, S., Gotthard, G., Bonvalet, A., Coquelle, N., Samire, P., Aleksandrov, A., Antonucci, L., Benachir, A., Boutet, S., Byrdin, M., Cammarata, M., Carbajo, S., Cuine, S., Doak, R. B., Foucar, L., Gorel, A., Grunbein, M., Hartmann, E., Hienerwadel, R., Hilpert, M., Kloos, M., Lane, T. J., Legeret, B., Legrand, P., Li-Beisson, Y., Moulin, S. L. Y., Nurizzo, D., Peltier, G., Schiro, G., Shoeman, R. L., Sliwa, M., Solinas, X., Zhuang, B., Barends, T. R. M., Colletier, J. P., Joffre, M., Royant, A., Berthomieu, C., Weik, M., Domratcheva, T., Brettel, K., Vos, M. H., Schlichting, I., Arnoux, P., Muller, P. & Beisson, F. (2021). *Science* **372**, eabd5687.

Stocker, S., Foschum, F., Krauter, P., Bergmann, F., Hohmann, A., Scalfi Happ, C. & Kienle, A. (2017). *Appl Spectrosc* **71**, 951-962.

Strack, D., Vogt, T. & Schliemann, W. (2003). *Phytochemistry* **62**, 247-269.

Wendel, M., Nizinski, S., Tuwalska, D., Starzak, K., Szot, D., Prukala, D., Sikorski, M., Wybraniec, S. & Burdzinski, G. (2015). *Phys. Chem. Chem. Phys.* **17**, 18152-18158.
